# Supplementary material for: Protective effects of Pt-N-C single-atom nanozymes against myocardial ischemia-reperfusion injury
Source: Nat Commun. 2024 Feb 23;15:1682. doi: 10.1038/s41467-024-45927-3 (PMC10891101; doi:10.1038/s41467-024-45927-3)
Supplement: Supplementary file 1 — Supplementary Information [file 41467_2024_45927_MOESM1_ESM.pdf]

SUPPLEMENTARY INFORMATION

Protective Effects of Pt-N-C Single-atom Nanozymes against Myocardial Ischemia-reperfusion Injury

Ye *et al*.

Supplementary Figures

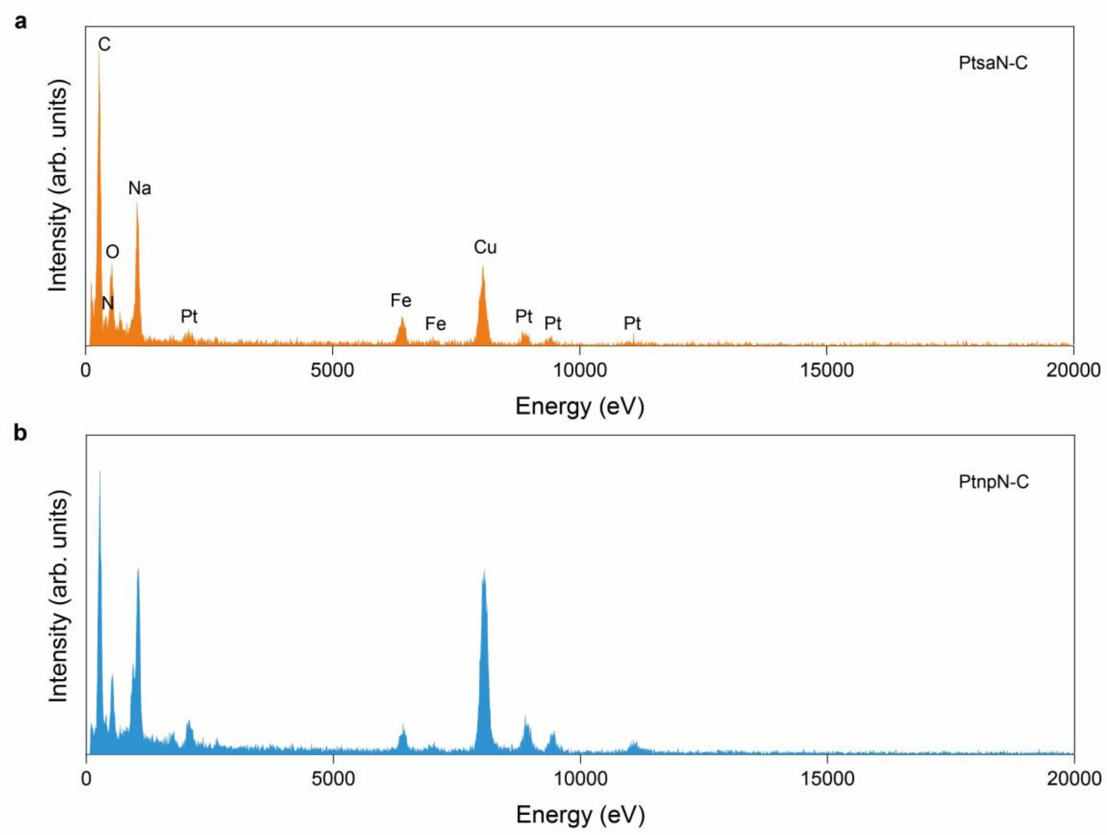

Supplementary Fig. 1 EDS spectrum of PtsaN-C (**a**) and PtnpN-C (**b**). Source data are provided as a Source Data file.

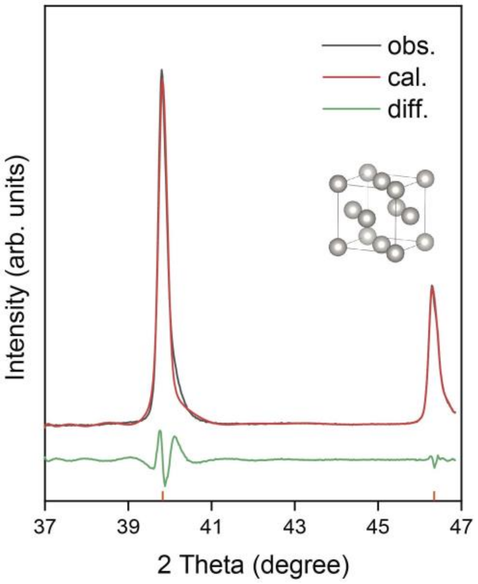

Supplementary Fig. 2 Rietveld-refined PtnpN-C catalyst. Inset was the calculated Pt cell unit. Source data are provided as a Source Data file.

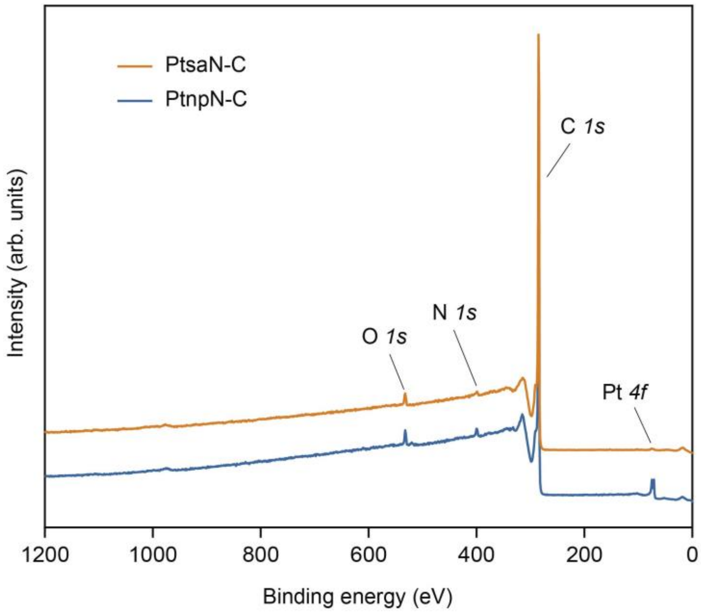

Supplementary Fig. 3 XPS survey spectra for pristine PtsaN-C and PtnpN-C. Source data are provided as a Source Data file.

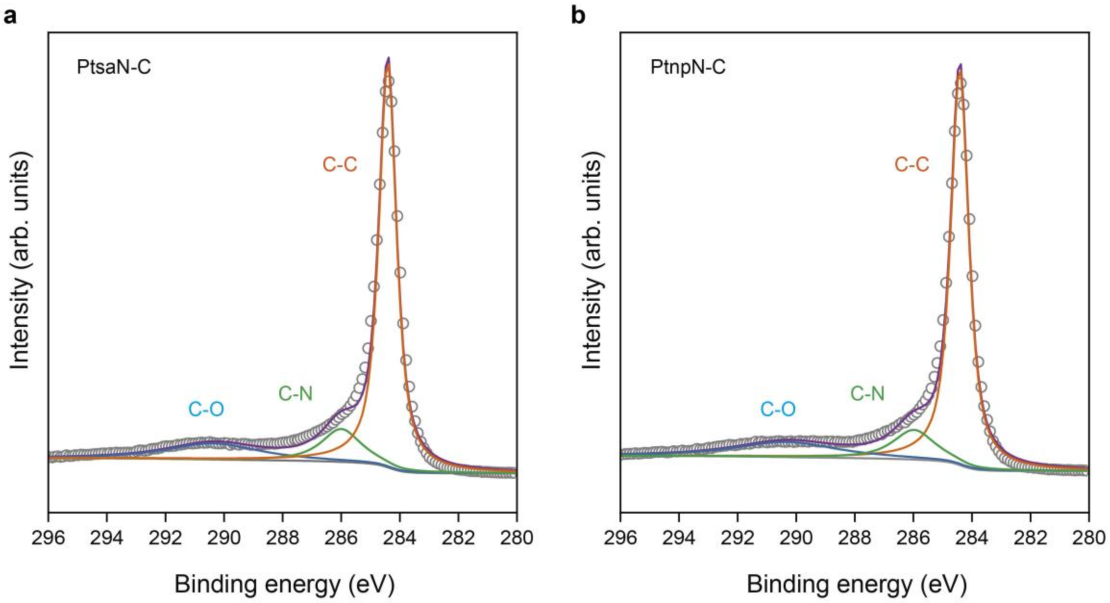

Supplementary Fig. 4 Core-level XPS spectra on C *1s* region for PtsaN-C (**a**) and PtnpN-C (**b**). Source data are provided as a Source Data file.

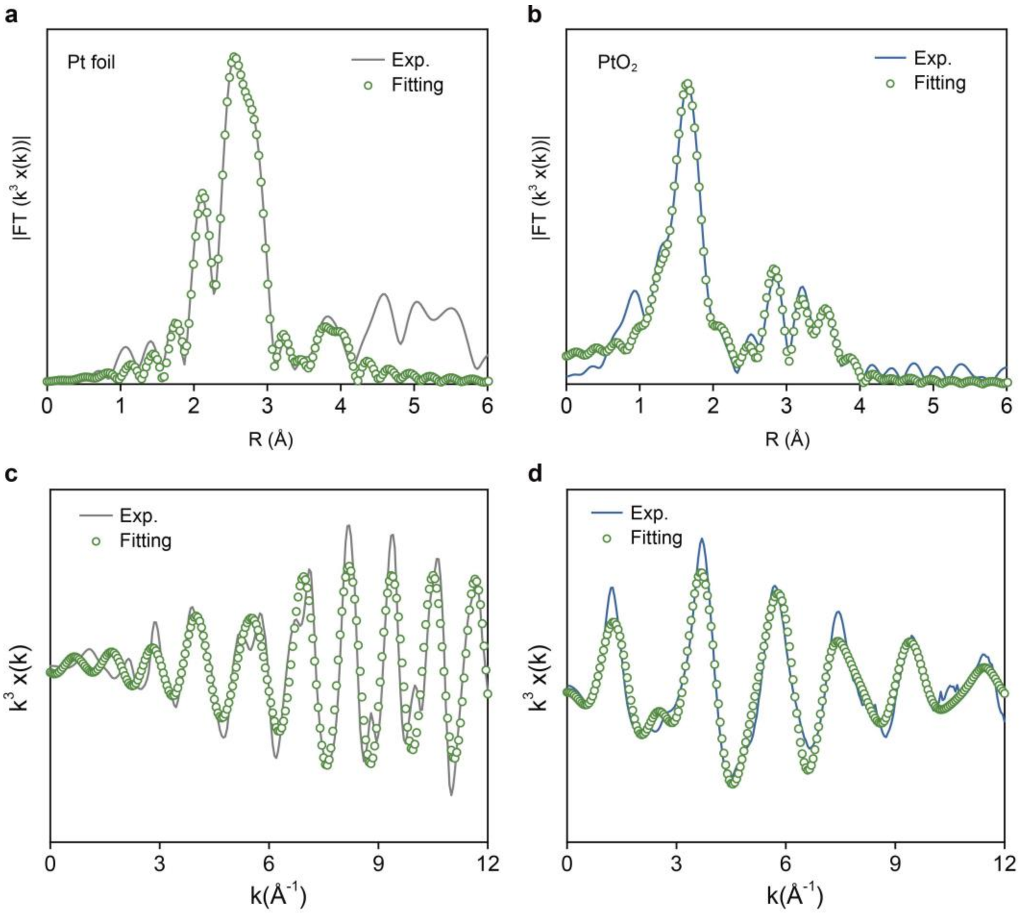

Supplementary Fig. 5 FT-EXAFS fitting curves of Pt (**a**) and PtO_2 (**b**) at the Pt L3-edge using Pt-Pt path and Pt-O path, respectively. The k-space fitting results of Pt (**c**) and PtO_2 (**d**). Source data are provided as a Source Data file.

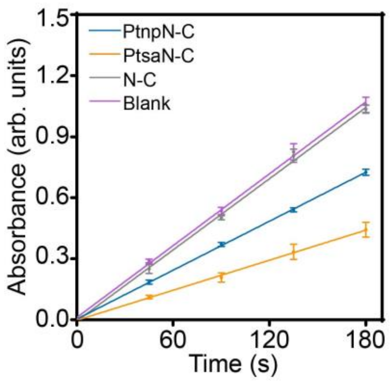

Supplementary Fig. 6 Time-dependent SOD-like activity was characterized by the absorbance changes of identifiable formazan (n = 3 independent samples). Data are presented with mean ± SEM. Source data are provided as a Source Data file.

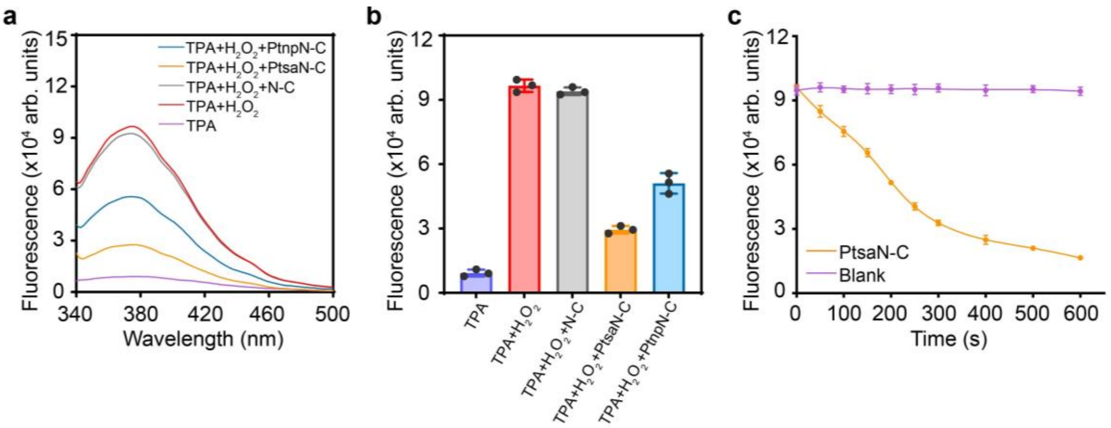

Supplementary Fig. 7 CAT-like activity of PtsaN-C detected by TPA. a Fluorescent spectrum of PtsaN-C, PtnpN-C, N-C, and TPA reference samples. Experiments were repeated three times with similar results. **b** Corresponding fluorescent values of indicated groups at peak wavelength (n = 3 independent samples). **c** Time-dependent fluorescent changes in the presence of H_2O_2 and TPA with or without PtsaN-C (n = 3 independent samples). Data are presented with mean ± SEM. Source data are provided as a Source Data file.

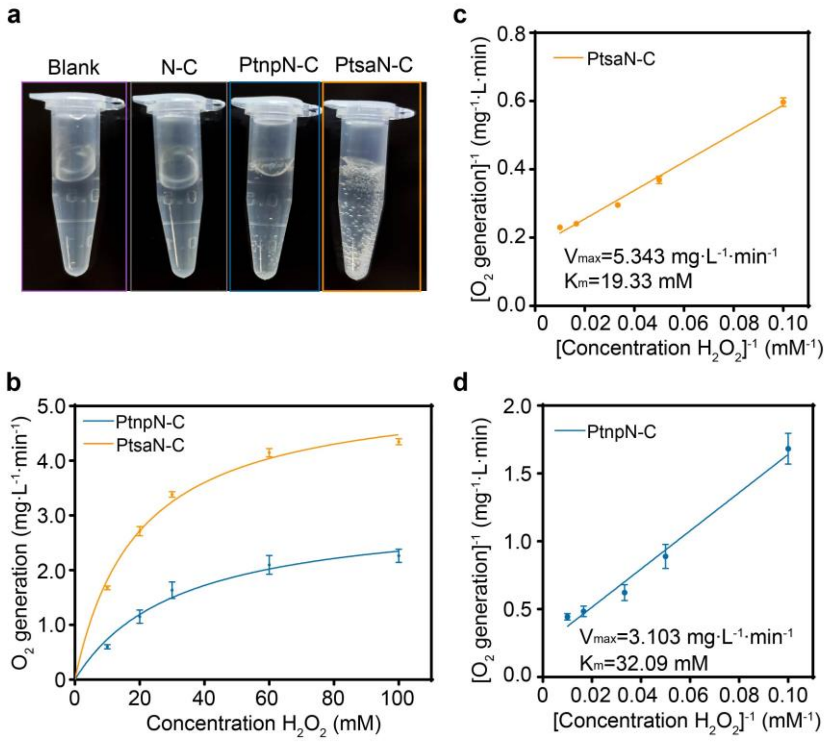

Supplementary Fig. 8 CAT-like activity of PtsaN-C detected by O_2 generation. a Digital picture of the bubble generation, indicating the decomposition of H_2O_2 into O_2. Experiments were repeated three times with similar results. **b** Steady-state kinetics analysis of PtsaN-C and PtnpN-C. (n = 3 independent samples). **c,d** Lineweaver–Burk plots of CAT-like activity of PtsaN-C **(c)** and PtnpN-C **(d)** corresponding to the variation of H_2O_2 concentration (n = 3 independent samples). Data are presented with mean ± SEM. Source data are provided as a Source Data file.

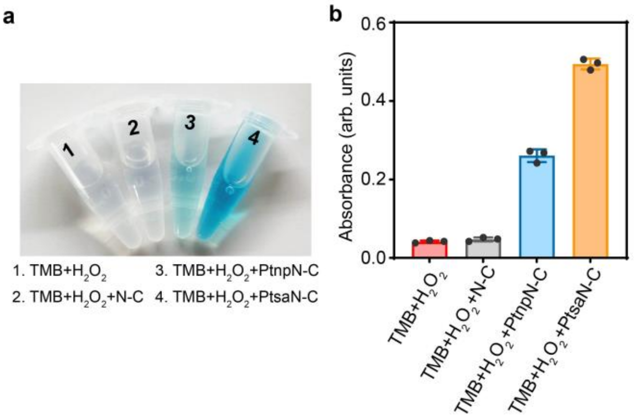

Supplementary Fig 9. POD-like activity of PtsaN-C. a Digital photograph of the color changes at different reaction solutions. Experiments were repeated three times with similar results. **b** Corresponding absorbance values of TMB for different treatments at 652 nm (n = 3 independent samples). Data are presented with mean ± SEM. Source data are provided as a Source Data file.

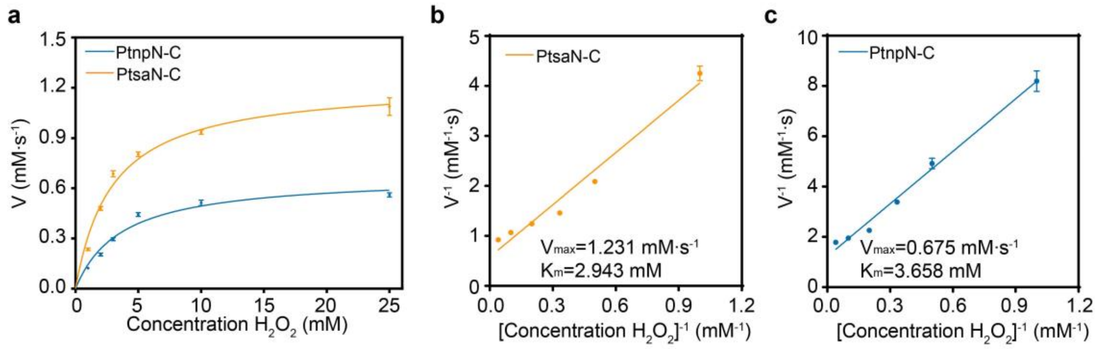

Supplementary Fig. 10 Michaelis-Menten kinetic analysis of POD-like activity of PtsaN-C. a Steady-state kinetics analysis of PtsaN-C and PtnpN-C against H_2O_2 substrate (n = 3 independent samples). **b, c** Corresponding Lineweaver–Burk plots of POD-like activity of PtnpN-C **(b)** and PtsaN-C **(c)** against the variation of H_2O_2 concentration (n = 3 independent samples). Data are presented with mean ± SEM. Source data are provided as a Source Data file.

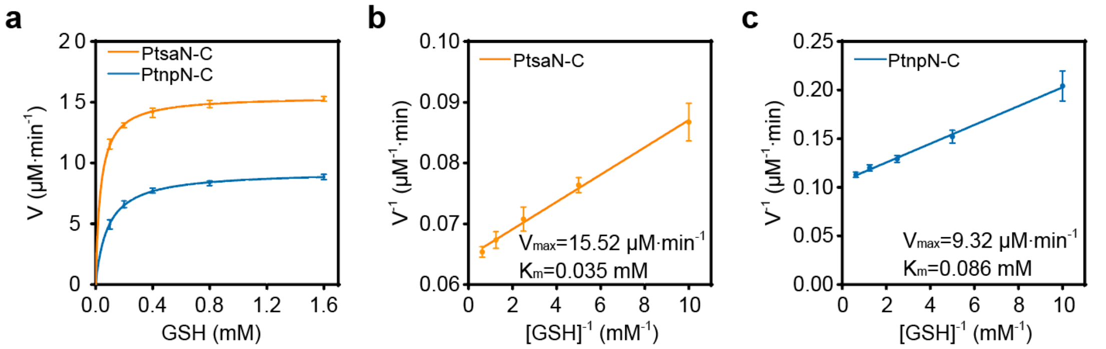

Supplementary Fig. 11 GPx-like activity of PtsaN-C analyzed by Michaelis-Menten kinetic. **a** Steady-state kinetics analysis of PtsaN-C and PtnpN-C against GSH substrate (n = 3 independent samples). **b**, **c** Corresponding Lineweaver–Burk plots of GPx-like activity of PtsaN-C (**b**) and PtnpN-C (**c**) against the variation of GSH concentration (n = 3 independent samples). Data are presented with mean ± SEM. Source data are provided as a Source Data file.

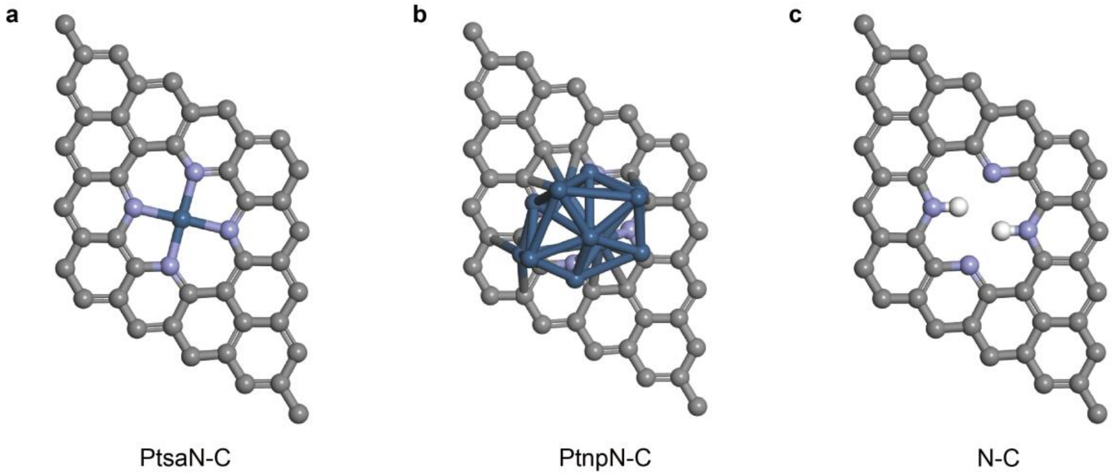

Supplementary Fig. 12 Illustration of PtsaN-C (**a**), PtnpN-C (**b**) and N-C (**c**).

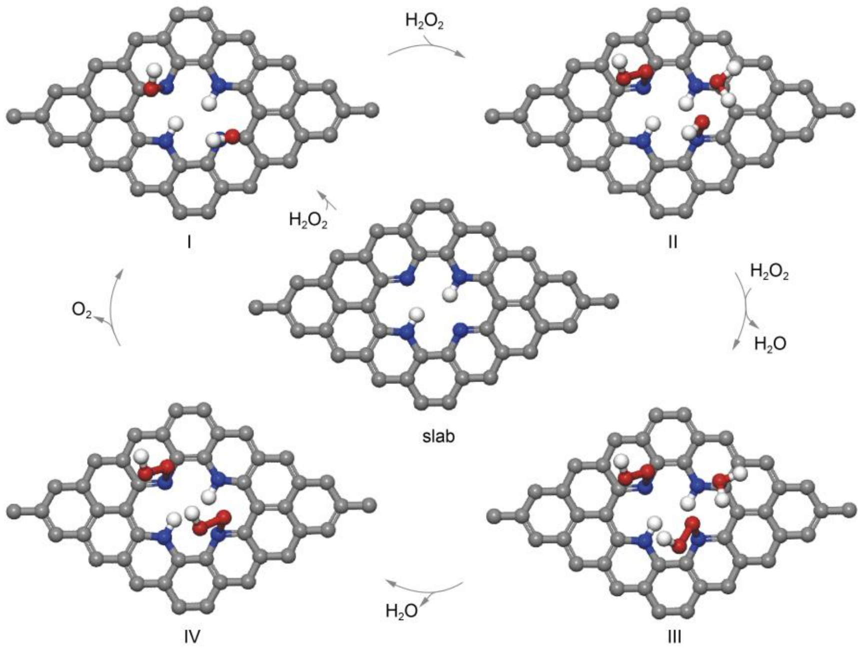

Supplementary Fig. 13 Proposed reaction pathways on N-C model.

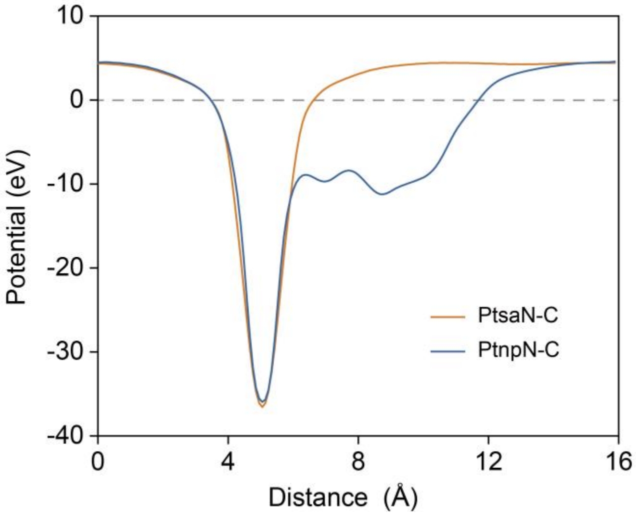

Supplementary Fig. 14 Calculated potential along *Z* axis of PtsaN-C and PtnpN-C. Fermi level was set as 0. Source data are provided as a Source Data file.

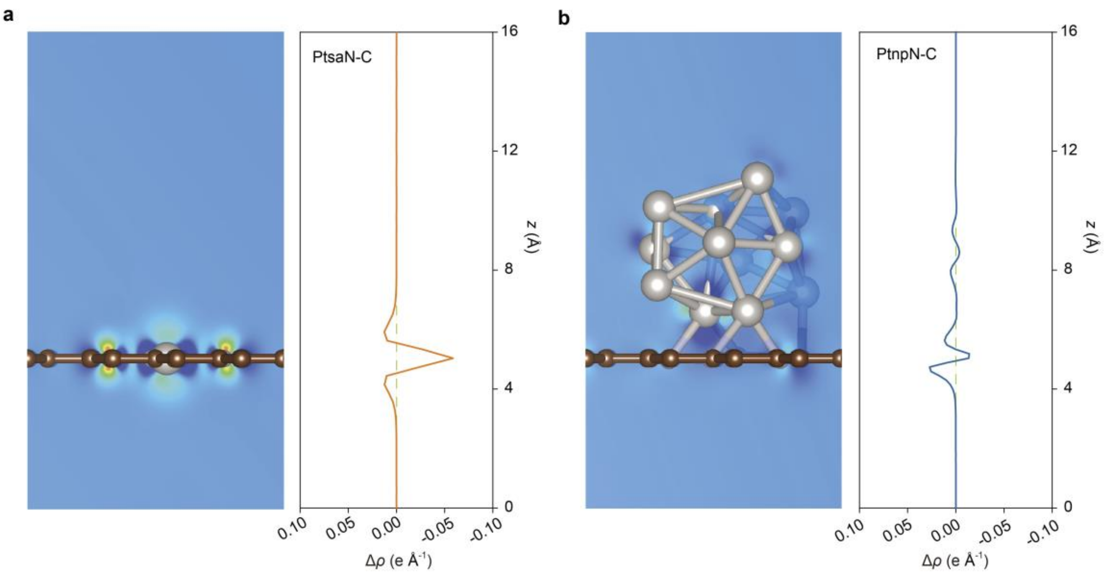

Supplementary Fig. 15 Two-dimensional and one-dimensional charge density difference on PtsaN-C (**a**) and PtnpN-C (**b**) (cyan and yellow represent charge depletion and accumulation, respectively). Source data are provided as a Source Data file.

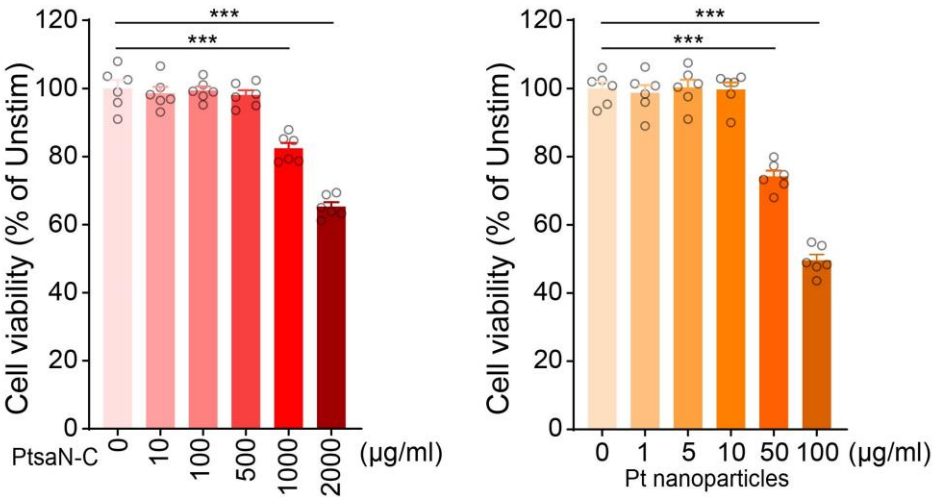

Supplementary Fig. 16 The cytotoxicity of the PtsaN-C and Pt nanoparticles. Cell viability was obtained at different concentrations of PtsaN-C (**left**) and bare Pt nanoparticles (**right**) in H9C2 cells using CCK-8 kit (n = 6 for biologically independent samples; PtsaN-C, $^{***}P_{(0,\ 1000)} < 0.0001$, $^{***}P_{(0,\ 2000)} < 0.0001$; Pt nanoparticles, $^{***}P_{(0,\ 50)} < 0.0001$, $^{***}P_{(0,\ 100)} < 0.0001$). Data are analyzed with One-way ANOVA with Bonferroni post hoc test, and presented with mean ± SEM. Source data are provided as a Source Data file.

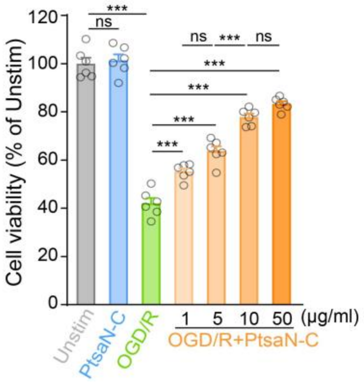

Supplementary Fig. 17 PtsaN-C enhancing cell viability during OGD/R procedure. The cell viability was accessed after OGD/R process in H9C2 cells with different concentration of PtsaN-C (n = 6 for biologically independent samples; $^{ns}P_{(\text{Unstim, PtsaN-C})} > 0.9999$, $^{***}P_{(\text{Unstim, OGD/R})} < 0.0001$, $^{***}P_{(\text{OGD/R, 1})} = 0.0006$, $^{***}P_{(\text{OGD/R, 5})} < 0.0001$, $^{***}P_{(\text{OGD/R, 10})} < 0.0001$, $^{***}P_{(\text{OGD/R, 50})} < 0.0001$, $^{ns}P_{(1,\ 5)} < 0.0850$, $^{***}P_{(5,\ 10)} = 0.0002$, $^{ns}P_{(10,\ 50)} > 0.9999$). Data are analyzed with One-way ANOVA with Bonferroni post hoc test, and presented with mean ± SEM. ns, no significance. Source data are provided as a Source Data file.

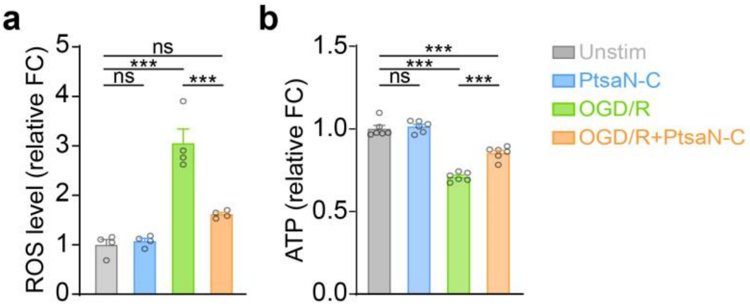

Supplementary Fig. 18 PtsaN-C mitigated ROS during OGD/R process. a Quantification of cellular ROS level in cells with different disposes (n = 4 for biologically independent samples; $^{ns}P_{(\text{Unstim, PtsaN-C})} > 0.9999$, $^{***}P_{(\text{Unstim, OGD/R})} < 0.0001$, $^{ns}P_{(\text{Unstim, OGD/R+PtsaN-C})} = 0.1067$, $^{***}P_{(\text{OGD/R, OGD/R+PtsaN-C})} = 0.0002$). **b** Cellular ATP content for indicated groups (n = 6 for biologically independent samples; $^{ns}P_{(\text{Unstim, PtsaN-C})} > 0.9999$, $^{***}P_{(\text{Unstim, OGD/R})} < 0.0001$, $^{***}P_{(\text{Unstim, OGD/R+PtsaN-C})} < 0.0001$, $^{***}P_{(\text{OGD/R, OGD/R+PtsaN-C})} < 0.0001$). Data are analyzed with One-way ANOVA with Bonferroni post hoc test, and presented with mean ± SEM. ns, no significance; FC, fold change. Source data are provided as a Source Data file.

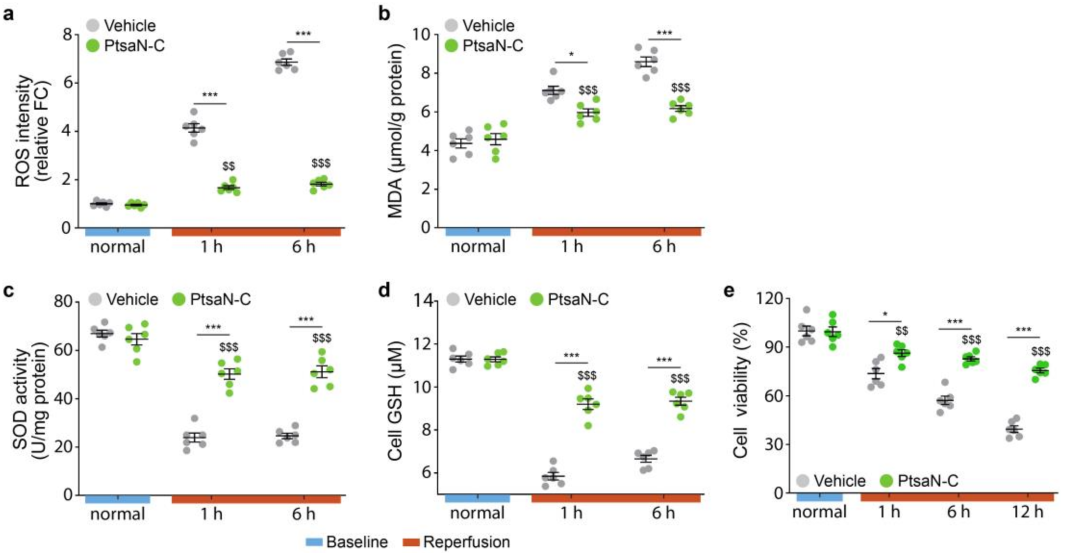

Supplementary Fig. 19 ROS decomposition of PtsaN-C over time. **a** ROS measured by ROS Assay Kit at the indicated time (n = 6 for biologically independent samples; $^{***}P_{(1h)} < 0.0001$, $^{***}P_{(6h)} < 0.0001$, $^{\$\$}P_{(1h)} = 0.0011$, $^{\$\$\$}P_{(6h)} < 0.0001$). **b-d** The cellular MDA (**b**), SOD (**c**), and GSH (**d**) levels in each group at different times after reoxygenation (n = 6 for biologically independent samples; **b**, $^{*}P_{(1h)} = 0.0171$, $^{***}P_{(6h)} < 0.0001$, $^{\$\$\$}P_{(1h)} = 0.0004$, $^{\$\$\$}P_{(6h)} < 0.0001$; **c**, $^{***}P_{(1h)} < 0.0001$, $^{***}P_{(6h)} < 0.0001$, $^{\$\$\$}P_{(1h)} < 0.0001$, $^{\$\$\$}P_{(6h)} < 0.0001$; **d**, $^{***}P_{(1h)} < 0.0001$, $^{***}P_{(6h)} < 0.0001$, $^{\$\$\$}P_{(1h)} = 0.0004$, $^{\$\$\$}P_{(6h)} < 0.0001$). **e** Cell viability during OGD/R injury (n = 6 for biologically independent samples; $^{*}P_{(1h)} = 0.0171$, $^{***}P_{(6h)} < 0.0001$, $^{***}P_{(12h)} < 0.0001$, $^{\$\$}P_{(1h)} = 0.0074$, $^{\$\$\$}P_{(6h)} = 0.0003$, $^{\$\$\$}P_{(12h)} < 0.0001$). Cells were treated with 10μg/ml PtsaN-C or equivalents of vehicle. Data are analyzed with Two-way ANOVA with Bonferroni post hoc test, and presented with mean ± SEM. $ is presented the comparison between PtsaN-C and normal Vehicle groups. FC, fold change. Source data are provided as a Source Data file.

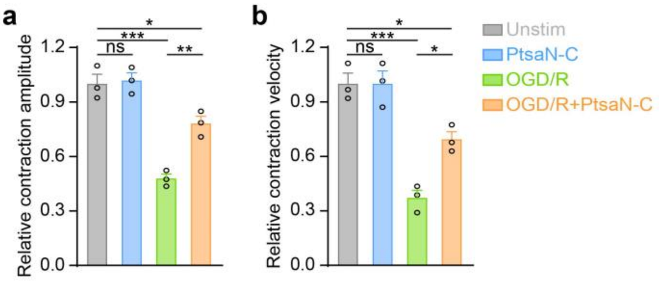

Supplementary Fig. 20 PtsaN-C restored cardiomyocyte contractile activity after the OGD/R procedure. a Quantitative analysis of the cardiomyocyte contraction amplitude (n = 3 for biologically independent samples; $^{ns}P_{(Unstim,\ PtsaN\text{-}C)} > 0.9999$, $^{***}P_{(Unstim,\ OGD/R)} = 0.0001$, $^{*}P_{(Unstim,\ OGD/R+PtsaN\text{-}C)} = 0.0336$, $^{**}P_{(OGD/R,\ OGD/R+PtsaN\text{-}C)} = 0.0050$). **b** Quantification of the cardiomyocyte contraction velocity (n = 3 for biologically independent samples; $^{ns}P_{(Unstim,\ PtsaN\text{-}C)} > 0.9999$, $^{***}P_{(Unstim,\ OGD/R)} = 0.0002$, $^{*}P_{(Unstim,\ OGD/R+PtsaN\text{-}C)} = 0.0250$, $^{*}P_{(OGD/R,\ OGD/R+PtsaN\text{-}C)} = 0.0187$). Data are analyzed with One-way ANOVA with Bonferroni post hoc test, and presented with mean ± SEM. ns, no significance. Source data are provided as a Source Data file.

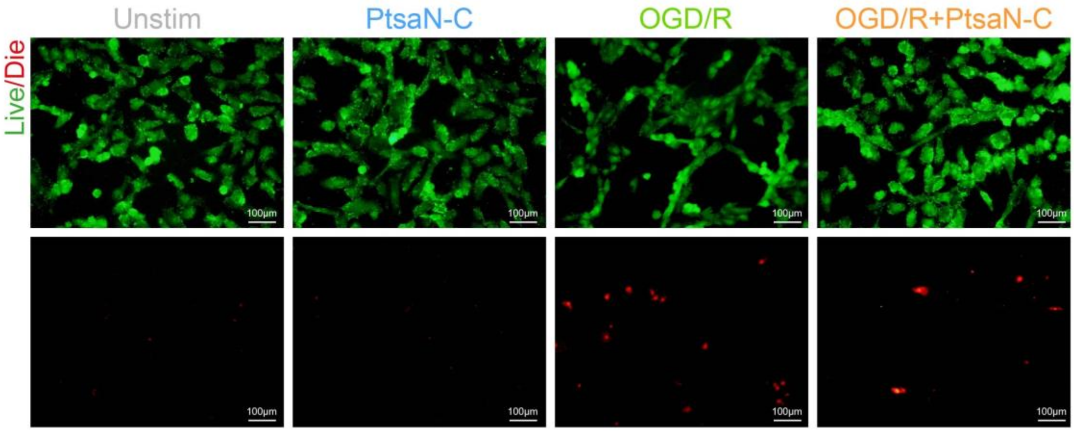

Supplementary Fig. 21 Representative fluorescence images of H9C2 cells under various experimental conditions. The cells were stained by calcein-AM/PI. Experiments were repeated three times with similar results. Scale bar: 100 μm.

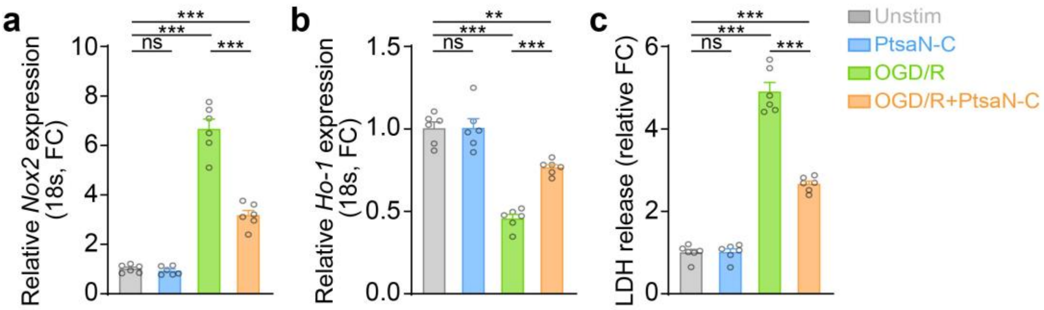

Supplementary Fig. 22 PtsaN-C alleviated apoptosis and cell injury during the OGD/R process. a,b Relative mRNA expression of Nox2 (**a**) and Ho-1 (**b**) in cells with different treatments (n = 6 for biologically independent samples; **a**, ${}^{ns}P_{(\text{Unstim, PtsaN-C})} > 0.9999$, ${}^{***}P_{(\text{Unstim, OGD/R})} < 0.0001$, ${}^{***}P_{(\text{Unstim, OGD/R+PtsaN-C})} < 0.0001$, ${}^{***}P_{(\text{OGD/R, OGD/R+PtsaN-C})} < 0.0001$; **b**, ${}^{ns}P_{(\text{Unstim, PtsaN-C})} > 0.9999$, ${}^{***}P_{(\text{Unstim, OGD/R})} < 0.0001$, ${}^{**}P_{(\text{Unstim, OGD/R+PtsaN-C})} = 0.0012$, ${}^{***}P_{(\text{OGD/R, OGD/R+PtsaN-C})} < 0.0001$). **c** Quantification of LDH levels (n = 6 for biologically independent samples; ${}^{ns}P_{(\text{Unstim, PtsaN-C})} > 0.9999$, ${}^{***}P_{(\text{Unstim, OGD/R})} < 0.0001$, ${}^{***}P_{(\text{Unstim, OGD/R+PtsaN-C})} < 0.0001$, ${}^{***}P_{(\text{OGD/R, OGD/R+PtsaN-C})} < 0.0001$). Data are analyzed with One-way ANOVA with Bonferroni post hoc test, and presented with mean ± SEM. ns, no significance; FC, fold change. Source data are provided as a Source Data file.

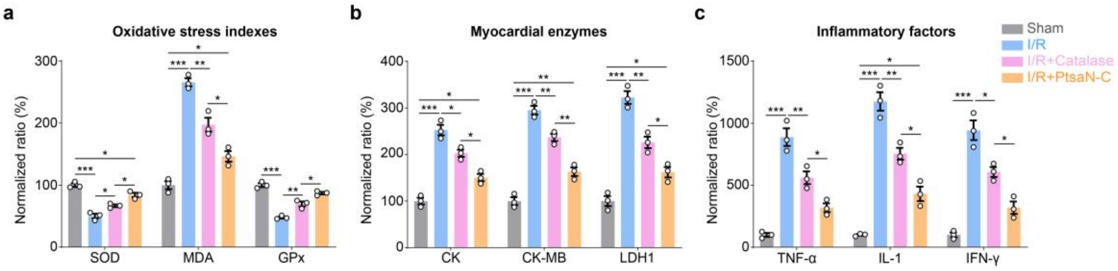

Supplementary Fig. 23 PtsaN-C showed an obvious advantage over Catalase on ischemia/reperfusion injury, in terms of levels of oxidative stress (**a**), myocardial enzymes (**b**), and inflammatory factors (**c**) (n = 3 for biologically independent animals; SOD, $^{***}P_{(Sham,\ I/R)} < 0.0001$, $^{*}P_{(Sham,\ I/R+PtsaN\text{-}C)} = 0.0417$, $^{*}P_{(I/R,\ I/R+Catalase)} = 0.0316$, $^{*}P_{(I/R+Catalase,\ I/R+PtsaN\text{-}C)} = 0.0255$; MDA, $^{***}P_{(Sham,\ I/R)} < 0.0001$, $^{*}P_{(Sham,\ I/R+PtsaN\text{-}C)} = 0.0265$, $^{**}P_{(I/R,\ I/R+Catalase)} = 0.0025$, $^{*}P_{(I/R+Catalase,\ I/R+PtsaN\text{-}C)} = 0.0150$; GPx, $^{***}P_{(Sham,\ I/R)} < 0.0001$, $^{**}P_{(I/R,\ I/R+Catalase)} = 0.0033$, $^{*}P_{(I/R+Catalase,\ I/R+PtsaN\text{-}C)} = 0.0133$; CK, $^{***}P_{(Sham,\ I/R)} < 0.0001$, $^{*}P_{(Sham,\ I/R+PtsaN\text{-}C)} = 0.0197$, $^{*}P_{(I/R,\ I/R+Catalase)} = 0.0210$, $^{*}P_{(I/R+Catalase,\ I/R+PtsaN\text{-}C)} = 0.0169$; CK-MB, $^{***}P_{(Sham,\ I/R)} < 0.0001$, $^{**}P_{(Sham,\ I/R+PtsaN\text{-}C)} = 0.0056$, $^{**}P_{(I/R,\ I/R+Catalase)} = 0.0091$, $^{**}P_{(I/R+Catalase,\ I/R+PtsaN\text{-}C)} = 0.0020$; LDH1, $^{***}P_{(Sham,\ I/R)} < 0.0001$, $^{*}P_{(Sham,\ I/R+PtsaN\text{-}C)} = 0.0409$, $^{**}P_{(I/R,\ I/R+Catalase)} = 0.0029$, $^{*}P_{(I/R+Catalase,\ I/R+PtsaN\text{-}C)} = 0.0337$; TNF-α, $^{***}P_{(Sham,\ I/R)} < 0.0001$, $^{**}P_{(I/R,\ I/R+Catalase)} = 0.0077$, $^{*}P_{(I/R+Catalase,\ I/R+PtsaN\text{-}C)} = 0.0441$; IL-1, $^{***}P_{(Sham,\ I/R)} < 0.0001$, $^{*}P_{(Sham,\ I/R+PtsaN\text{-}C)} = 0.0134$, $^{**}P_{(I/R,\ I/R+Catalase)} = 0.0030$, $^{*}P_{(I/R+Catalase,\ I/R+PtsaN\text{-}C)} = 0.0154$; INF-γ, $^{***}P_{(Sham,\ I/R)} < 0.0001$, $^{*}P_{(I/R,\ I/R+Catalase)} = 0.0102$, $^{*}P_{(I/R+Catalase,\ I/R+PtsaN\text{-}C)} = 0.0179$). Data are analyzed with One-way ANOVA with Bonferroni post hoc test, and presented with mean ± SEM. Source data are provided as a Source Data file.

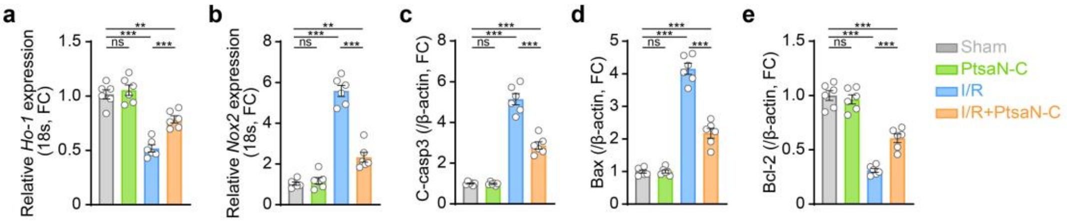

Supplementary Fig. 24 The antiapoptotic effect of PtsaN-C on ischemia/reperfusion heart. a,b Expression levels of Ho-1 (**a**) and Nox2 (**b**) mRNAs, normalized to 18s mRNA (n = 6 for biologically independent animals; **a**, $^{ns}P_{(Sham,\ PtsaN\text{-}C)} > 0.9999$, $^{***}P_{(Sham,\ I/R)} < 0.0001$, $^{**}P_{(Sham,\ I/R+PtsaN\text{-}C)} = 0.0033$, $^{***}P_{(I/R,\ I/R+PtsaN\text{-}C)} = 0.0007$; **b**, $^{ns}P_{(Sham,\ PtsaN\text{-}C)} > 0.9999$, $^{***}P_{(Sham,\ I/R)} < 0.0001$, $^{**}P_{(Sham,\ I/R+PtsaN\text{-}C)} = 0.0013$, $^{***}P_{(I/R,\ I/R+PtsaN\text{-}C)} < 0.0001$). **c-e** Quantitative densitometry of the indicated proteins related to Fig. 7k (n = 6 for biologically independent animals; **c**, $^{ns}P_{(Sham,\ PtsaN\text{-}C)} > 0.9999$, $^{***}P_{(Sham,\ I/R)} < 0.0001$, $^{***}P_{(Sham,\ I/R+PtsaN\text{-}C)} < 0.0001$, $^{***}P_{(I/R,\ I/R+PtsaN\text{-}C)} < 0.0001$; **d**, $^{ns}P_{(Sham,\ PtsaN\text{-}C)} > 0.9999$, $^{***}P_{(Sham,\ I/R)} < 0.0001$, $^{***}P_{(Sham,\ I/R+PtsaN\text{-}C)} < 0.0001$, $^{***}P_{(I/R,\ I/R+PtsaN\text{-}C)} < 0.0001$; **e**, $^{ns}P_{(Sham,\ PtsaN\text{-}C)} > 0.9999$, $^{***}P_{(Sham,\ I/R)} < 0.0001$, $^{***}P_{(Sham,\ I/R+PtsaN\text{-}C)} < 0.0001$, $^{***}P_{(I/R,\ I/R+PtsaN\text{-}C)} = 0.0001$). Data are presented with mean ± SEM and analyzed with One-way ANOVA with Bonferroni post hoc test. ns, no significance; FC, fold change. Source data are provided as a Source Data file.

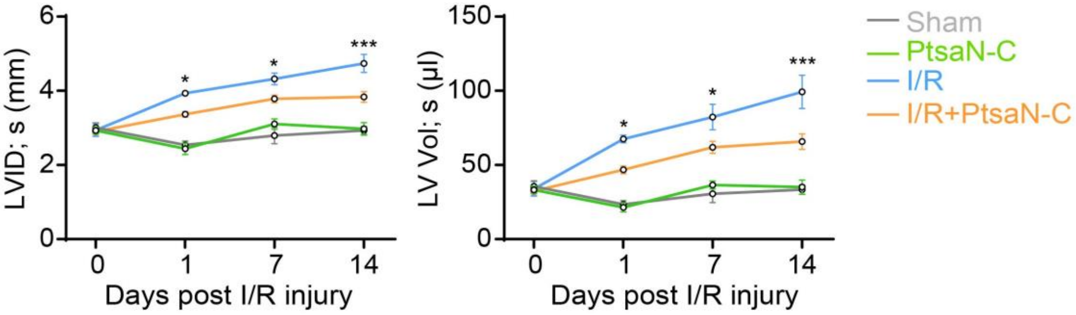

Supplementary Fig. 25 Cardiac function measured by LVID and LV volume at systolic in cardiac long-axis (comparison between I/R and I/R + PtsaN-C; LVID: $^{*}P_{(1d)} = 0.0122$, $^{*}P_{(7d)} = 0.0203$, $^{***}P_{(14d)} < 0.0001$; LV Vol: $^{*}P_{(1d)} = 0.0206$, $^{*}P_{(7d)} = 0.0247$, $^{***}P_{(14d)} < 0.0001$). Data are presented with mean ± SEM and analyzed with Two-way ANOVA with Bonferroni post hoc test. Source data are provided as a Source Data file.

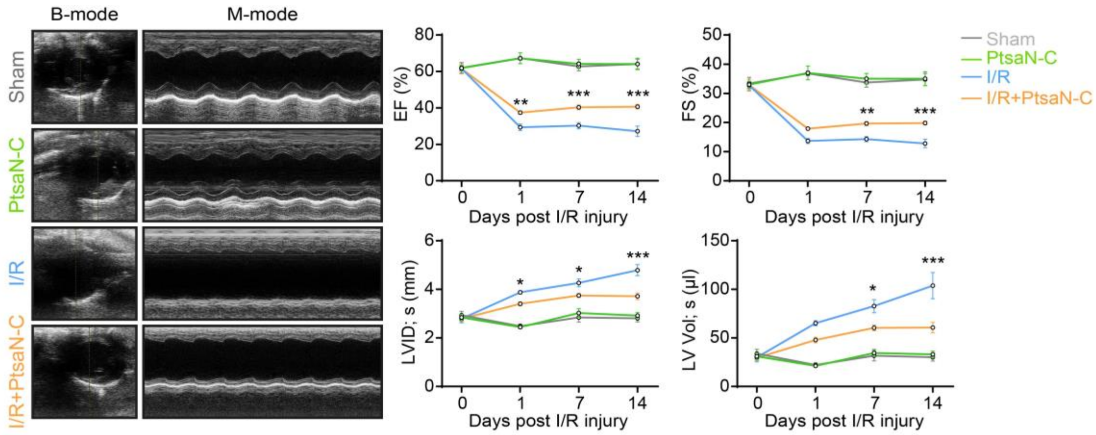

Supplementary Fig. 26 PtsaN-C improved cardiac function measured in cardiac short-axis (comparison between I/R and I/R + PtsaN-C; EF: $^{**}P_{(1d)} = 0.0078$, $^{***}P_{(7d)} = 0.0005$, $^{***}P_{(14d)} < 0.0001$; FS: $^{**}P_{(7d)} = 0.0059$, $^{***}P_{(14d)} = 0.0001$; LVID: $^{*}P_{(1d)} = 0.0383$, $^{*}P_{(7d)} = 0.0178$, $^{***}P_{(14d)} < 0.0001$; LV Vol: $^{*}P_{(7d)} = 0.0138$, $^{***}P_{(14d)} < 0.0001$). Data are presented with mean ± SEM and analyzed with Two-way ANOVA with Bonferroni post hoc test. Source data are provided as a Source Data file.

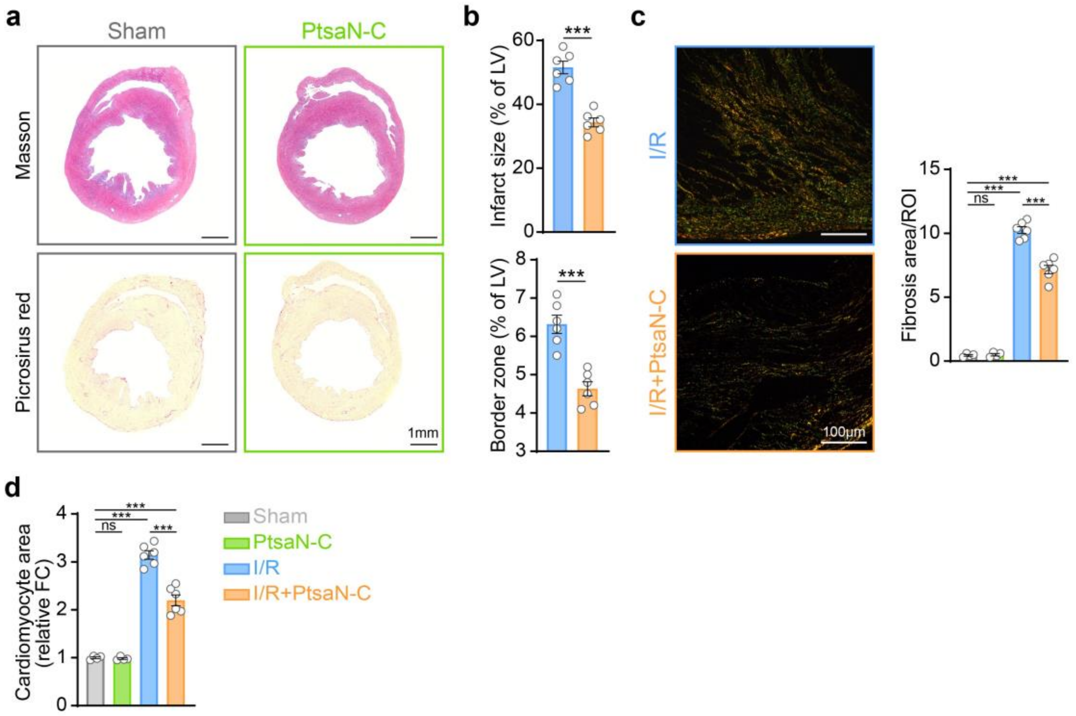

Supplementary Fig. 27 PtsaN-C reduced infarct size, myocardial fibrosis, and cardiomyocyte hypertrophy. a Presentative Masson trichrome staining, Picrosirius red staining of Sham and PtsaN-C Sham group. Experiments were repeated four times with similar results. Scale bar: 1 mm. **b** Quantification of infarct size, and border zone transition followed by pipeline analysis (n = 6 for biologically independent animals; Infarct size, $^{***}P$ < 0.0001; Border zone, $^{***}P$ = 0.0002). **c** Picrosirius red staining imaged under polarized light. Quantification is shown right. (n = 4 biologically independent animals for Sham groups and 6 for I/R groups; $^{ns}P_{(\text{Sham, PtsaN-C})}$ > 0.9999, $^{***}P_{(\text{Sham, I/R})}$ < 0.0001, $^{***}P_{(\text{Sham, I/R+PtsaN-C})}$ < 0.0001, $^{***}P_{(\text{I/R, I/R+PtsaN-C})}$ < 0.0001; scale bar: 100 μm) **d** Quantification of cross-sectional area of the cardiomyocytes in border zone 14 days post I/R injury (n = 4 biologically independent animals for Sham groups and 6 for I/R groups; $^{ns}P_{(\text{Sham, PtsaN-C})}$ > 0.9999, $^{***}P_{(\text{Sham, I/R})}$ < 0.0001, $^{***}P_{(\text{Sham, I/R+PtsaN-C})}$ < 0.0001, $^{***}P_{(\text{I/R, I/R+PtsaN-C})}$ < 0.0001)). Data are presented with mean ± SEM and analyzed with two-tailed unpaired Student t-test (**b**), One-way ANOVA with Bonferroni post hoc test (**c**, **d**). ns, no significance; FC, fold change; LV, left ventricle; ROI, region of interest. Source data are provided as a Source Data file.

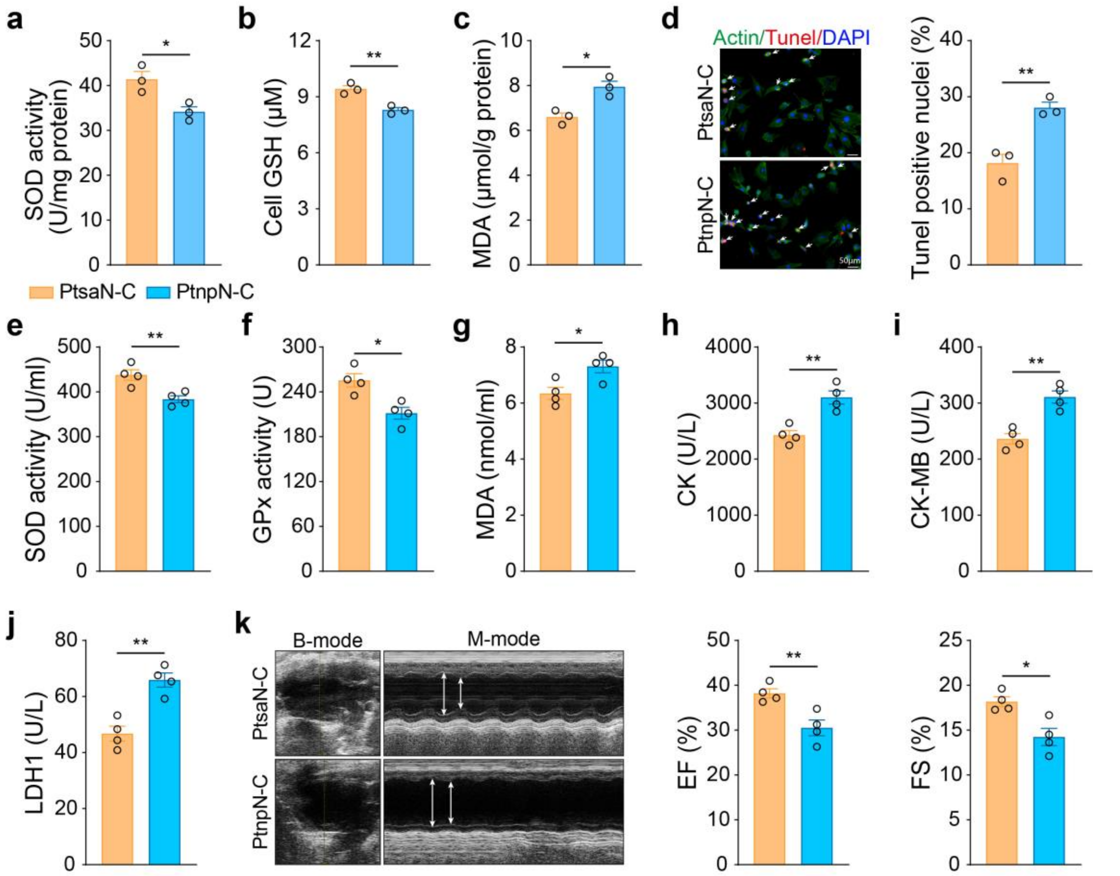

Supplementary Fig. 28 PtsaN-C represents a distinct therapeutic advantage over PtnpN-C. **a-c** The levels of cellular SOD (**a**), GSH (**b**), and MDA (**c**) measured after OGD/R process (n = 3 for biologically independent samples; **a**, $^{*}P$ = 0.0257; **b**, $^{**}P$ = 0.0061; **c**, $^{*}P$ = 0.0125). **d** Apoptosis of cells detected by Tunel assay after OGD/R process (n = 3 for biologically independent samples; $^{**}P$ = 0.0063; scale bar: 50 μm). **e-g** The levels of SOD (**e**), GPx (**f**), MDA (**g**) in cardiac tissue homogenate at day 1 after I/R injury (n = 4 for biologically independent animals; **e**, $^{**}P$ = 0.0084; **f**, $^{*}P$ = 0.0100; **g**, $^{*}P$ = 0.0216). **h-j** Serum concentrations of myocardial enzyme spectrum CK (**h**), CK-MB (**i**), and LDH1 (**j**) tested at day 1 after I/R injury (n = 4 for biologically independent animals; **h**, $^{**}P$ = 0.0034; **i**, $^{**}P$ = 0.0020; **j**, $^{**}P$ = 0.0020). **k** Cardiac function measured by echocardiography at day 1 after I/R injury (n = 4 for biologically independent animals; EF, $^{**}P$ = 0.0090; FS, $^{*}P$ = 0.0114). Data are represented with the mean ± SEM and analyzed with two-tailed unpaired Student t-test. Source data are provided as a Source Data file.

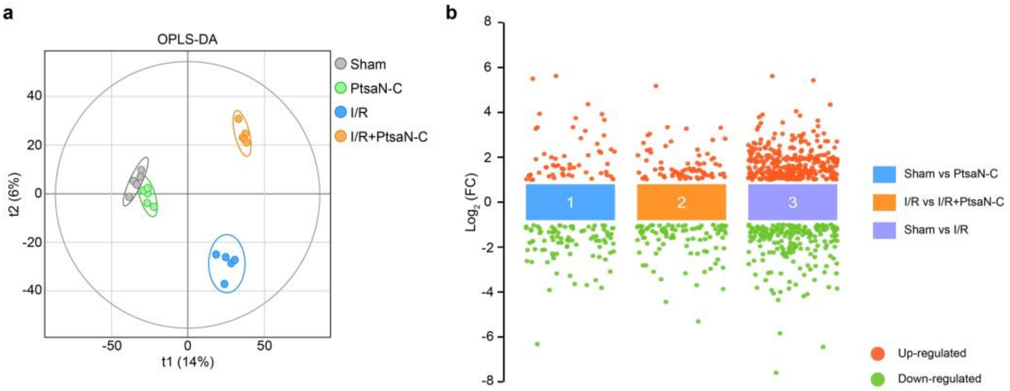

Supplementary Fig. 29 a Orthogonal projections to latent structures discriminant analysis (OPLS-DA) scores showed similar expression profiles between Sham and PtsaN-C groups, indicating the histocompatibility of PtsaN-C. Obvious differences were seen among Sham, I/R, and I/R+PtsaN-C groups. **b** Volcano plots showing differentially expressed proteins between indicated groups, and each point represents a protein. Data are analyzed with moderated two-tailed t-test (Limma). Source data are provided as a Source Data file.

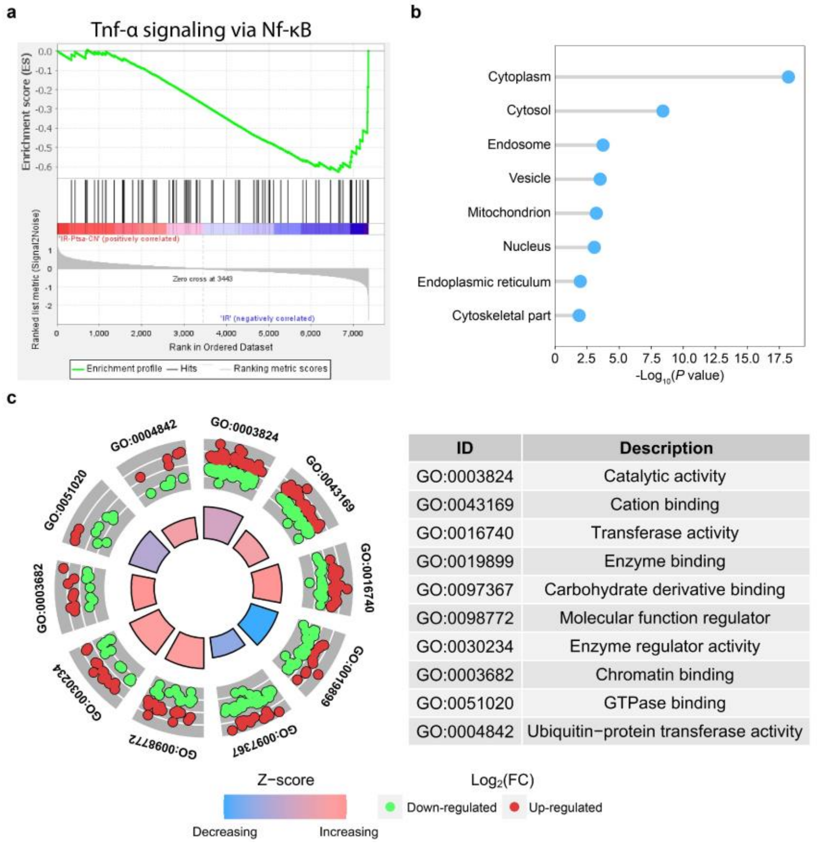

Supplementary Fig. 30 a GSEA analysis revealed Tnf-α signaling via NF-kb was highly activated in I/R group compared to PtsaN-C treatment (Empirical phenotype-based permutation test, $P < 0.001$, NSE = -1.61, FDR = 0.122). **b** Cell component (CC) referred to GO terms of differentially expressed proteins (Two-tailed Fisher's exact test). **c** Enrichment of molecular function (MF) referred to GO terms of differentially expressed proteins. Source data are provided as a Source Data file.

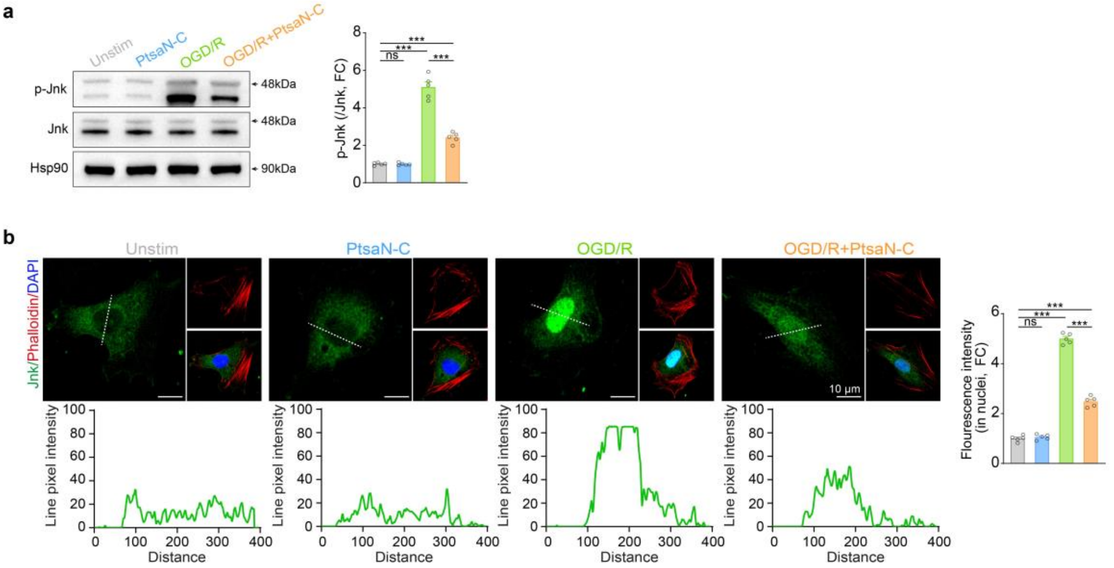

Supplementary Fig. 31 PtsaN-C depresses the activation of MAPK/Jnk pathway induced by I/R injury. a Representative western blot of p-Jnk in cells with different disposes and corresponding quantification (n = 5 for biologically independent samples; $^{ns}P_{(\mathrm{Unstim,\ PtsaN\text{-}C})} > 0.9999$, $^{***}P_{(\mathrm{Unstim,\ OGD/R})} < 0.0001$, $^{***}P_{(\mathrm{Unstim,\ OGD/R+PtsaN\text{-}C})} < 0.0001$, $^{***}P_{(\mathrm{OGD/R,\ OGD/R+PtsaN\text{-}C})} < 0.0001$). **b** Results of immunofluorescent cell staining analysis of Jnk, and relative quantification of flourescence intensity in nuclei (n = 5 for biologically independent samples; $^{ns}P_{(\mathrm{Unstim,\ PtsaN\text{-}C})} > 0.9999$, $^{***}P_{(\mathrm{Unstim,\ OGD/R})} < 0.0001$, $^{***}P_{(\mathrm{Unstim,\ OGD/R+PtsaN\text{-}C})} < 0.0001$, $^{***}P_{(\mathrm{OGD/R,\ OGD/R+PtsaN\text{-}C})} < 0.0001$; scale bar: 10 μm). Data are presented with mean ± SEM and analyzed with One-way ANOVA with Bonferroni post hoc test. ns, no significance; FC, fold change. Source data are provided as a Source Data file.

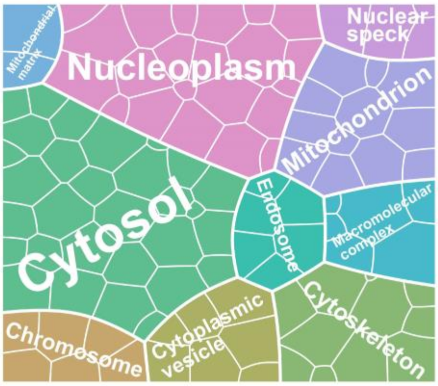

Supplementary Fig. 32 Cell component enrichment terms of differentially expressed proteins between Sham and PtsaN-C groups.

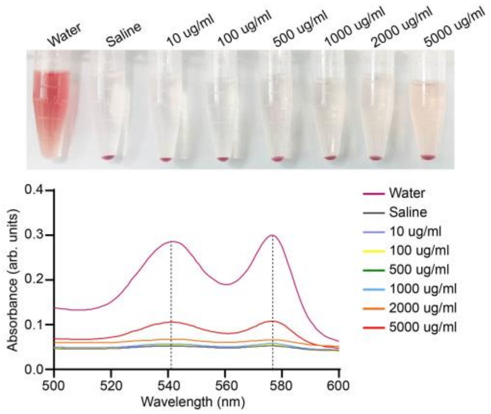

Supplementary Fig. 33 The haematological biocompatibility of PtsaN-C was carried out using a haemolytic experiment in vitro. Fresh blood cells were incubated with various concentrations of PtsaN-C for 20min. Photographs (upper) were taken and hemoglobin absorbance spectrum (down) was measured. Experiments were repeated three times with similar results. Source data are provided as a Source Data file.

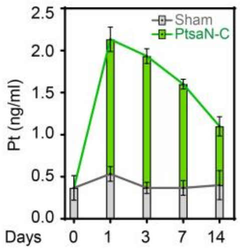

Supplementary Fig. 34 Platinum metal levels in urine were determined by ICP-MS at indicated time points (n = 3 for biologically independent animals). Data are presented with mean ± SEM. Source data are provided as a Source Data file.

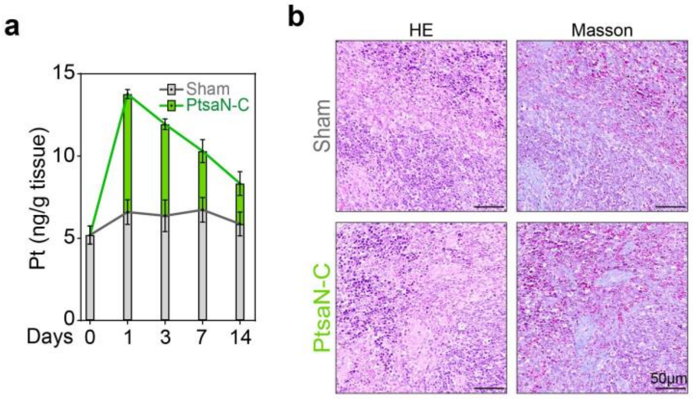

Supplementary Fig. 35 a ICP-MS analysis of spleen at indicated time points (n = 3 for biologically independent animals). **b** In vivo evaluation of the toxicity of PtsaN-C to spleen by histological analysis of HE and Masson trichrome staining at day 14 post-administration. Experiments were repeated three times with similar results. Scale bar: 50 μm. Data are presented with mean ± SEM. Source data are provided as a Source Data file.

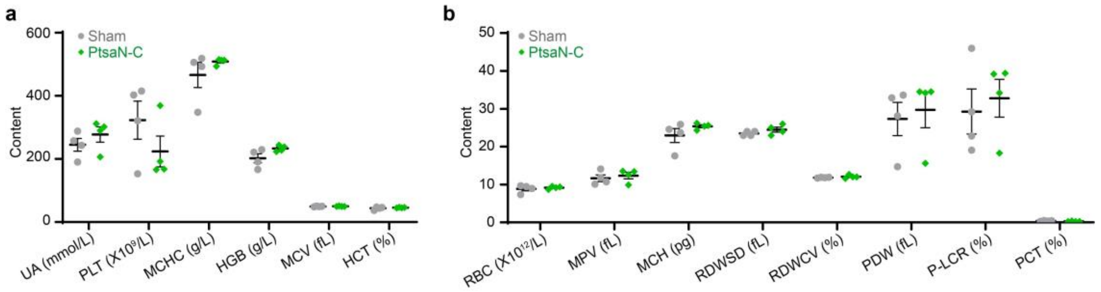

Supplementary Fig. 36 In vivo biosafety of PtsaN-C. Complete blood panel analysis and UA concentration results in mice after injection with PtsaN-C (n = 4 for biologically independent animals). Data are presented with mean ± SEM with two-tailed unpaired Student t-test. Source data are provided as a Source Data file.

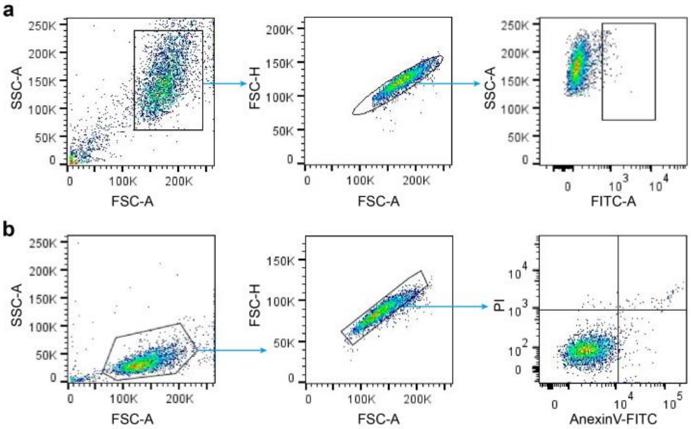

Supplementary Fig. 37 Gating strategies of flow cytometry. a Gating strategies of intracellular ROS levels in **Fig. 6b**. **b** Gating strategies for cellular apoptosis in **Fig. 6g**.

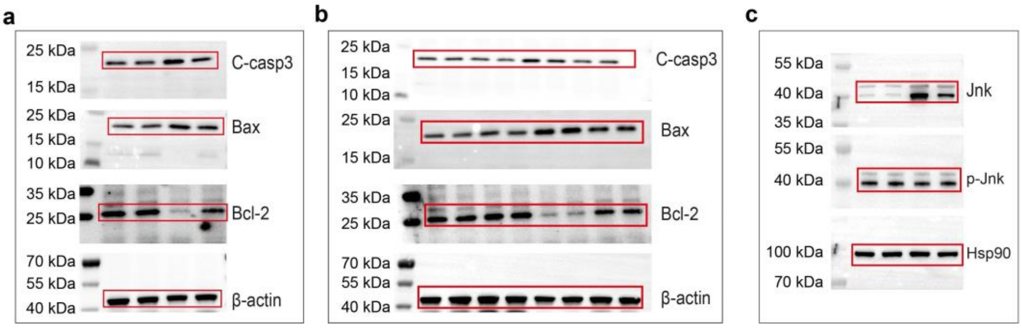

Supplementary Fig. 38 The respective original images of western blot. a The original western blot images presented in **Fig. 6l**. **b** The original western blot images presented in **Fig. 7k**. **c** The original western blot images presented in **Supplementary Fig. 31a**.

Supplementary Tables

Supplementary Table 1. ICP-OES and XPS results for Pt and N contents respectively in different samples.

Sample	PtsaN-C	PtnpN-C
Pt (wt%)	0.78	3.76
N (wt%)	2.31	2.05

Supplementary Table 2. Structural information, including space group, lattice parameters, discrepancy factor (Rwp), and goodness fit factor (χ^2), derived from the Rietveld refinement of PtnpN-C powder XRD spectra and Pt (JCPDS: 04-0802) standards.

Sample	Space group	Cell length (Å)	Cell angle (°)	Cell volume (Å^3)	R_{wp} (%)	χ^2
PtnpN-C	Fm-3m	a: 3.910<br>b: 3.910<br>c: 3.910	a: 90<br>b: 90<br>c: 90	59.8	12.24	15.44
Pt (JCPDS: 04-0802)		a: 3.944<br>b: 3.944<br>c: 3.944	a: 90<br>b: 90<br>c: 90	61.3	-	-

Supplementary Table 3. Chemical environment analysis of Pt measured by XPS

Sample	PtsaN-C	PtnpN-C
Pt^{2+} (%)	100	36.5
Pt^{0} (%)	0	63.5

Supplementary Table 4. Chemical environment analysis of N measured by XPS

Sample	PtsaN-C	PtnpN-C
Pyridinic N (%)	73.3	28.4
Graphitic N (%)	26.7	71.6

Supplementary Table 5. EXAFS fitting parameters at the Pt L3-edge for various samples ($S0^2$=0.715).

Sample	Shell	*CN*[a]	*R*(Å)[b]	σ^2 ($Å^2$) [c]	ΔE_0 (eV)[d]	R factor (%)
Pt foil	Pt-Pt	11.76±0.71	2.76±0.04	0.004	8.25±0.52	1.3
PtO_2	Pt-O	5.68±0.40	2.02±0.01	0.002	10.563±1.48	0.67
	Pt-O	3.42±0.58	3.20±0.01	0.002		
	Pt-Pt	3.55±1.71	3.52±0.04	0.013		
PtsaN-C	Pt-N	3.95±0.73	2.05±0.05	0.008	9.07±1.18	1.47

[a]CN, coordination number; [b]R, distance between absorber and backscatter atoms; [c]σ^2, Debye-Waller factor to account for both thermal and structural disorders; [d]ΔE0, inner potential correction; R factor indicates the goodness of the fit. Fitting range: 2.5 < k ($Å^{-1}$) < 11.0 and 1.0 < R (Å)< 3.0 (Pt foil); 2.5 < k($Å^{-1}$) <10.0 and 1.0 < R(Å) < 4.0 (PtO_2); 2.5 < k(Å $^{-1}$) < 10.0 and 1.0<R(Å)< 3.0 (PtsaN-C).

Supplementary Table 6. Comparison of kinetic parameters with recently reported nanozymes with CAT-like catalysis.

Nanozymes	K_m (mM)	V_{max} (μM s^{-1})	[E] (μM)	TON (s^{-1})	Ref.
Fe_1NC	380.00	1.01	0.627 (ICP-OES)	1.6108	Adv. Funct. Mater. 2022, 32, 2204025.
Fe_2NC	110.00	2.71	0.681 (ICP-OES)	3.9794	
Pt-iNOS@ZIF	172.00	3.46	0.236 (ICP-OES)	14.6610	Nat Commun. 2022, 13, 2513.
$Au_{24}Cu_1$	392.00	5.83	3.000 (ICP-MS)	1.9433	Nat Commun. 2021, 12, 114.
Pero-nanozysome (Fe)	90.00	1.22	4.820 (ICP-OES)	0.2531	Adv. Funct. Mater. 2021, 31, 2007130.
SAuPTB	1.50	0.38	74.631 (ICP-MS)	0.0051	Small. 2023, 19, e2206408
Ru_{SA}-CN	330.15	2.07	0.930 (ICP-OES)	2.2258	Adv Mater. 2022, 34, e2206208
Ru_{NC}-CN	225.76	19.61	1.630 (ICP-OES)	12.0307	
Ru_{NP}-CN	246.97	14.64	2.310 (ICP-OES)	6.3377	
Fe^{3+}/AMP CPs	112.20	2.40	0.800 (ICP-OES)	3.0000	Chem. Eng. J. 2020, 388, 124249.
Multi-caged IrOx NPs	187.95	5.64	520.000 (ICP-OES)	0.0108	Angew Chem Int Ed Engl. 2020,59, 9491-9497
MoO_x-Cu-Cys-PVP Sazymes	2.02	4.75	79.470 (ICP-MS)	0.0598	J. Am. Chem. Soc. 2023, 145, 4279–4293
RhN4	1.33	0.50	0.056 (ICP-MS)	8.9286	Nat Commun. 2022, 13, 4744
VN4	2.92	0.15	0.128 (ICP-MS)	1.1719	
FeN4	2.04	0.92	0.750 (ICP-MS)	1.2267	
CuN4	3.17	0.32	0.657 (ICP-MS)	0.4871	
Fe-Cu-N6	1.99	1.05	0.555 (ICP-MS)	1.8919	
BSA-IrO_2 NPs	206.19	1.86	520.000 (ICP-OES)	0.0036	Adv. Funct. Mater. 2020, 30, 2002274.
Multi-caged IrO_x NPs	145.81	5.72	520.000 (ICP-OES)	0.0110	
Catalase	52.31	2.41	-	-	
PtsaN-C	19.33	2.78	0.200 (ICP-OES)	13.9057	This work
PtnpN-C	32.09	1.62	0.964 (ICP-OES)	1.6805	This work

[E], the molar concentration of the metal activation sites in nanozymes detected by ICP-OES or ICP-MS; K_m, Michaelis constant; V_{max}, maximal reaction velocity; TON, turnover number (the catalytic constant), where TON = V_{max}/[E].

Supplementary Table 7. The kinetic energy barriers for the dissociation of three H_2O_2 molecules on the PtsaN-C, PtnpN-C, and N-C models.

Model	Step	Reaction	Energy barrier(eV)
PtsaN-C	Slab→I	$*H_2O_2 \rightarrow 2*OH$	0.10
	I→II	$*OH + H_2O_2 \rightarrow *OOH + *H_2O$	0.57
	II→III	$*OH + H_2O_2 \rightarrow *OOH + *H_2O$	0.19
PtnpN-C	Slab→I	$*H_2O_2 \rightarrow 2*OH$	0.17
	I→II	$*OH + H_2O_2 \rightarrow *OOH + *H_2O$	0.90
	II→III	$*OH + H_2O_2 \rightarrow *OOH + *H_2O$	0.29
N-C	Slab→I	$*H_2O_2 \rightarrow 2*OH$	0.20
	I→II	$*OH + H_2O_2 \rightarrow *OOH + *H_2O$	1.61
	II→III	$*OH + H_2O_2 \rightarrow *OOH + *H_2O$	0.14

Supplementary Table 8. Sum of echocardiography

Day post-I/R	0d				1d				7d				14d			
**Group**	**Sham**	**PtsaN-C**	**I/R**	**I/R+PtsaN-C**	**Sham**	**PtsaN-C**	**I/R**	**I/R+PtsaN-C**	**Sham**	**PtsaN-C**	**I/R**	**I/R+PtsaN-C**	**Sham**	**PtsaN-C**	**I/R**	**I/R+PtsaN-C**
**n**	5	5	5	5	5	5	8	10	5	5	8	10	5	5	8	10
**Age (Weeks)**	6-8	6-8	6-8	6-8	6-8	6-8	6-8	6-8	7-9	7-9	7-9	7-9	8-10	8-10	8-10	8-10
**Short-axis M-mode**																
**EF (%)**	62.01±2.89	61.85±1.54	61.59±1.48	61.5±2.82	67.3±0.96	67.28±2.97	29.37±1.77	37.45±0.89	62.76±2.39	64.15±2.48	30.25±1.69	40.32±1.27	64.06±2.72	64.13±3.13	27.21±2.82	40.69±1.18
**FS (%)**	33.36±2.14	33.01±1.14	32.75±0.98	32.83±2.04	36.83±0.81	37.01±2.35	13.66±0.87	17.87±0.48	33.74±1.64	35.02±1.81	14.29±0.89	19.64±0.7	34.78±2.08	35.01±2.38	12.82±1.41	19.83±0.65
**LVID; s (mm)**	2.94±0.16	2.84±0.13	2.79±0.16	2.79±0.12	2.48±0.1	2.45±0.06	3.88±0.07	3.4±0.08	2.84±0.19	3.03±0.18	4.27±0.16	3.75±0.08	2.8±0.14	2.92±0.12	4.79±0.23	3.71±0.13
**LV Vol; s (µl)**	33.82±4.38	31.14±3.36	29.96±4.56	29.52±2.92	22.15±2.19	21.23±1.37	65.16±2.63	47.94±2.78	31.64±5.3	34.56±3.7	82.74±6.8	60.38±2.97	30.17±3.99	33.08±3.31	103.83±13.46	60.73±5.3
**Long-axis M-mode**																
**EF (%)**	61.26±2.69	59.86±1.8	59.57±1.04	59.45±2.42	68.28±1.52	66.53±3.13	29.22±1.62	38.7±1.13	62.6±3.7	62.94±2.05	29.61±2.05	39.85±1.62	62.32±2.83	62.24±3.01	29.02±3.07	41.08±1.95
**FS (%)**	32.83±1.96	31.66±1.33	31.39±0.65	31.38±1.71	37.75±1.3	36.38±2.39	13.79±0.95	18.53±0.62	33.73±2.54	34.1±1.39	13.96±1.05	19.4±0.88	33.59±2.08	33.59±2.28	13.78±1.59	20.64±0.75
**LVID; s (mm)**	3±0.13	2.93±0.09	2.94±0.17	2.9±0.1	2.54±0.11	2.44±0.15	3.94±0.06	3.37±0.08	2.79±0.22	3.1±0.14	4.32±0.16	3.78±0.1	2.93±0.11	2.98±0.17	4.74±0.24	3.83±0.14
**LV Vol; s (µl)**	35.56±3.79	33.27±2.41	33.91±4.93	32.46±2.65	23.42±2.48	21.49±2.97	67.55±2.56	46.73±2.54	30.65±5.98	36.53±2.61	82.32±8.53	61.92±3.98	33.36±3.1	35.06±4.75	99.34±11.19	65.76±5.23
**HR (bpm)**	471.64±19.02	506.09±31.66	477.53±17.23	451.16±32.58	442.58±21.72	467.05±20.7	476.29±9.06	475.84±9.83	479.79±38.21	468.89±18.64	490.08±14.65	501.97±21.74	461.23±31.38	454.62±16.15	465.45±23.45	417.66±18.94

Data are presented as mean ± SEM. EF, ejection fraction; FS, fractional shortening; LVID; s, systolic left ventricular internal diameter; LV Vol; s, systolic left ventricular volume; HR, heart rate; bpm, beat per min.
