# Supplementary material for: Protective effects of Pt-N-C single-atom nanozymes against myocardial ischemia-reperfusion injury
Source: Nat Commun. 2024 Feb 23;15:1682. doi: 10.1038/s41467-024-45927-3 (PMC10891101; doi:10.1038/s41467-024-45927-3)
Supplement: Supplementary file 2 — Reporting Summary [file 41467_2024_45927_MOESM2_ESM.pdf]

nature portfolio

Corresponding author(s): Xianting Ding

Last updated by author(s): Feb 4, 2024

Reporting Summary

Nature Portfolio wishes to improve the reproducibility of the work that we publish. This form provides structure for consistency and transparency in reporting. For further information on Nature Portfolio policies, see our Editorial Policies and the Editorial Policy Checklist.

Statistics

For all statistical analyses, confirm that the following items are present in the figure legend, table legend, main text, or Methods section.

n/a	Confirmed	
☐	☒	The exact sample size (*n*) for each experimental group/condition, given as a discrete number and unit of measurement
☐	☒	A statement on whether measurements were taken from distinct samples or whether the same sample was measured repeatedly
☐	☒	The statistical test(s) used AND whether they are one- or two-sided<br>*Only common tests should be described solely by name; describe more complex techniques in the Methods section.*
☒	☐	A description of all covariates tested
☐	☒	A description of any assumptions or corrections, such as tests of normality and adjustment for multiple comparisons
☐	☒	A full description of the statistical parameters including central tendency (e.g. means) or other basic estimates (e.g. regression coefficient) AND variation (e.g. standard deviation) or associated estimates of uncertainty (e.g. confidence intervals)
☐	☒	For null hypothesis testing, the test statistic (e.g. *F*, *t*, *r*) with confidence intervals, effect sizes, degrees of freedom and *P* value noted<br>*Give P values as exact values whenever suitable.*
☒	☐	For Bayesian analysis, information on the choice of priors and Markov chain Monte Carlo settings
☒	☐	For hierarchical and complex designs, identification of the appropriate level for tests and full reporting of outcomes
☒	☐	Estimates of effect sizes (e.g. Cohen's *d*, Pearson's *r*), indicating how they were calculated

Our web collection on statistics for biologists contains articles on many of the points above.

Software and code

Policy information about availability of computer code

Data collection	Fluorescence microscope (Olympus IX-71); Flow cytometry (BD FACSCanto II); TEM (Tecnai G2 F20 S-Twin TEM); HAADF-STEM (ThermoFisher Themis Z microscope); XRD (Rigaku Mini Flex 600 spectrometer); XPS and UPS (Kratos AXIS Ultra DLD XPS); Confocal XploRA Raman spectrometer(XploRA); Echocardiography (Vevo 2100); Q-Exactive™ Plus Hybrid Quadrupole-Orbitrap™; ICP-MS (Agilent 7900 spectrometer); Microsoft Excel (version 2019); Chemiluminescence detection (TanonFine-DoX6); qRT-PCR (Roche LightCycler480 II); Epoch microplate reader (Biotech); NexION 5000 (Perkin Elmer); Leica Stellaris 5 (Leica); SpectraMax iD3 (Molecular Devices); Bruker A300 spectrometer; ICP-OES (ThermoFisher, iCAP 7600).
Data analysis	Microsoft Excel (version 2019); FlowJo (version 10.7.1); Origin 2021; GraphPad Prism 9.3.0; GSEA (version 4.1.0); Demeter (version 0.9.26); Material studio 2020; Image J software (version 1.52); Skyline (version 22.1); Cytoscape (version 3.8.2); GSEA (version 4.1.0).

For manuscripts utilizing custom algorithms or software that are central to the research but not yet described in published literature, software must be made available to editors and reviewers. We strongly encourage code deposition in a community repository (e.g. GitHub). See the Nature Portfolio guidelines for submitting code & software for further information.

Data

Policy information about availability of data

All manuscripts must include a data availability statement. This statement should provide the following information, where applicable:

- Accession codes, unique identifiers, or web links for publicly available datasets
- A description of any restrictions on data availability
- For clinical datasets or third party data, please ensure that the statement adheres to our policy

The mass spectrometry proteomics data generated in this study have been deposited in the ProteomeXchange Consortium under accession code PXD048584. All data needed to support the conclusions in the paper are available within the article and the Supplementary Information Files. Any other data related to this work are available from the corresponding author upon request. Source data are provided as a Source Data file in this paper.

Research involving human participants, their data, or biological material

Policy information about studies with human participants or human data. See also policy information about sex, gender (identity/presentation), and sexual orientation and race, ethnicity and racism.

Reporting on sex and gender	None.
Reporting on race, ethnicity, or other socially relevant groupings	None.
Population characteristics	None.
Recruitment	None.
Ethics oversight	None.

Note that full information on the approval of the study protocol must also be provided in the manuscript.

Field-specific reporting

Please select the one below that is the best fit for your research. If you are not sure, read the appropriate sections before making your selection.

[x] Life sciences [ ] Behavioural & social sciences [ ] Ecological, evolutionary & environmental sciences

For a reference copy of the document with all sections, see nature.com/documents/nr-reporting-summary-flat.pdf

Life sciences study design

All studies must disclose on these points even when the disclosure is negative.

Sample size	No statistical method was used to predetermine the sample size. The sample sizes were determined as minimal to lower the cost and be sufficient to obtain statistically significant difference between experimental groups. For in vivo studies, each group contains at least 3 for ensuring the statistical validity.
Data exclusions	No data was excluded in this study.
Replication	Experiments were repeated at least three times with similar results.
Randomization	Cells or mice were randomly assigned to different groups before treatments.
Blinding	Investigators were blinded to group allocation.

Reporting for specific materials, systems and methods

We require information from authors about some types of materials, experimental systems and methods used in many studies. Here, indicate whether each material, system or method listed is relevant to your study. If you are not sure if a list item applies to your research, read the appropriate section before selecting a response.

Materials & experimental systems

n/a	Involved in the study
	☒ Antibodies
	☒ Eukaryotic cell lines
☒	Palaeontology and archaeology
	☒ Animals and other organisms
☒	Clinical data
☒	Dual use research of concern
☒	Plants

Methods

n/a	Involved in the study
☒	ChIP-seq
	☒ Flow cytometry
☒	MRI-based neuroimaging

Antibodies

Antibodies used

Primary antibodies [format: host anti-protein (company, catalog number, dilution, Clone number if available, link)]
1. Mouse HRP-conjugated β-actin Monoclonal antibody (Proteintech, HRP-60008, 1:10000 for Westen-blot, 7D2C10, https://www.ptgcn.com/products/Beta-Actin-Antibody-HRP-60008.htm)
2. Mouse Hsp90 Monoclonal antibody (Proteintech, 60318-1-Ig, 1:5000 for Westen-blot, 3F11C1, https://www.ptgcn.com/products/HSP90-Antibody-60318-1-Ig.htm)
3. Rabbit anti-C-casp3 Polyclonal antibody (Proteintech, 25128-1-AP, 1:1000 for Westen-blot, https://www.ptgcn.com/products/cleaved-Caspase-3-Antibody-25128-1-AP.htm)
4. Mouse Bcl2 Monoclonal antibody (Proteintech, 68103-1-Ig, 1:2000 for Westen-blot, 1B3F7, https://www.ptgcn.com/products/Bcl2-Antibody-68103-1-Ig.htm)
5. Rabbit anti-Bax Monoclonal antibody (Abcam, ab32503, 1:1000 for Westen-blot, E63, https://www.abcam.cn/products/primary-antibodies/bax-antibody-e63-ab32503.html)
6. Rabbit JNK Polyclonal antibody (Proteintech, 24164-1-AP, 1:2000 for Westen-blot, https://www.ptgcn.com/products/JNK-Antibody-24164-1-AP.htm)
7. Rabbit JNK (phospho T183 + Y185) Monoclonal antibody (Abcam, ab307802, 1:1000 for Westen-blot, HL1008, https://www.abcam.cn/products/primary-antibodies/jnk-phospho-t183--y185-antibody-hl1008-ab307802.html)
8. Mouse cTnT Monoclonal antibody (Abcam, ab8295, 1:500 for Immunofluorescence, 1C11, https://www.abcam.cn/products/primary-antibodies/cardiac-troponin-t-antibody-1c11-ab8295.html)
9. Rabbit γ-H2AX Monoclonal antibody (Abcam, ab 81299, 1:200 for Immunofluorescence, EP854(2)Y, https://www.abcam.cn/products/primary-antibodies/gamma-h2ax-phospho-s139-antibody-ep8542y-ab81299.html)
10. Mouse β-actin Monoclonal antibody (Proteintech, 66009-1-Ig, 1:1000 for Immunofluorescence, 2D4H5, https://www.ptgcn.com/products/Pan-Actin-Antibody-66009-1-Ig.htm)

Secondary antibodies [format: host anti-protein (company, catalog number, dilution, lot number & Clone number if available)]
1. Goat HRP-conjugated Affinipure Goat Anti-Rabbit IgG(H+L) Polyclonal antibody (Proteintech, SA00001-2, 1:10000 for Westen-blot, https://www.ptgcn.com/products/HRP-conjugated-Affinipure-Goat-Anti-Rabbit-IgG-H-L-secondary-antibody.htm#product-information)
2. Goat HRP-conjugated Affinipure Goat Anti-Mouse IgG(H+L) Polyclonal antibody (Proteintech, SA00001-1, 1:10000 for Westen-blot, https://www.ptgcn.com/products/HRP-conjugated-Affinipure-Goat-Anti-Mouse-IgG-H-L-secondary-antibody.htm)
3. Goat anti-Rabbit IgG (H+L) Cross-Adsorbed Secondary Antibody, Alexa Fluor™ 594 (ThermoFisher, A-11012, 1:1000 for Immunofluorescence, https://www.thermofisher.cn/cn/zh/antibody/product/Goat-anti-Rabbit-IgG-H-L-Cross-Adsorbed-Secondary-Antibody-Polyclonal/A-11012)
4. Goat anti-Mouse IgG (H+L) Cross-Adsorbed Secondary Antibody, Alexa Fluor™ 488 (ThermoFisher, A-11001, 1:1000 for Immunofluorescence, https://www.thermofisher.cn/cn/zh/antibody/product/Goat-anti-Mouse-IgG-H-L-Cross-Adsorbed-Secondary-Antibody-Polyclonal/A-11001)
5. Goat anti-Mouse IgG (H+L) Cross-Adsorbed Secondary Antibody, Alexa Fluor™ 594 (ThermoFisher, A-11005, 1:1000 for Immunofluorescence, https://www.thermofisher.cn/cn/zh/antibody/product/Goat-anti-Mouse-IgG-H-L-Cross-Adsorbed-Secondary-Antibody-Polyclonal/A-11005)

Validation

All antibodies used in this study are commercially available, verified and quality-tested by the suppliers.
Primary antibodies
1. Mouse HRP-conjugated β-actin Monoclonal antibody (https://www.ptgcn.com/products/Beta-Actin-Antibody-HRP-60008.htm)
2. Mouse HSP90 Monoclonal antibody (https://www.ptgcn.com/products/HSP90-Antibody-60318-1-Ig.htm)
3. Rabbit anti-C-casp3 Polyclonal antibody (https://www.ptgcn.com/products/cleaved-Caspase-3-Antibody-25128-1-AP.htm)
4. Mouse Bcl2 Monoclonal antibody (https://www.ptgcn.com/products/Bcl2-Antibody-68103-1-Ig.htm)
5. Rabbit anti-Bax Monoclonal antibody (https://www.abcam.cn/products/primary-antibodies/bax-antibody-e63-ab32503.html)
6. Rabbit JNK Polyclonal antibody (https://www.ptgcn.com/products/JNK-Antibody-24164-1-AP.htm)
7. Rabbit JNK (phospho T183 + Y185) Monoclonal antibody (https://www.abcam.cn/products/primary-antibodies/jnk-phospho-t183--y185-antibody-hl1008-ab307802.html)
8. Mouse cTnT Monoclonal antibody (https://www.abcam.cn/products/primary-antibodies/cardiac-troponin-t-antibody-1c11-ab8295.html)
9. Rabbit γ-H2AX Monoclonal antibody (https://www.abcam.cn/products/primary-antibodies/gamma-h2ax-phospho-s139-antibody-ep8542y-ab81299.html)

10. Mouseβ-actin Monoclonal antibody (https://www.ptgcn.com/products/Pan-Actin-Antibody-66009-1-Ig.htm)

Secondary antibodies
1. Goat HRP-conjugated Affinipure Goat Anti-Rabbit IgG(H+L) Polyclonal antibody (https://www.ptgcn.com/products/HRP-conjugated-Affinipure-Goat-Anti-Rabbit-IgG-H-L-secondary-antibody.htm#product-information)
2. Goat HRP-conjugated Affinipure Goat Anti-Mouse IgG(H+L) Polyclonal antibody (https://www.ptgcn.com/products/HRP-conjugated-Affinipure-Goat-Anti-Mouse-IgG-H-L-secondary-antibody.htm)
3. Goat anti-Rabbit IgG (H+L) Cross-Adsorbed Secondary Antibody, Alexa Fluor™ 594 (https://www.thermofisher.cn/cn/zh/antibody/product/Goat-anti-Rabbit-IgG-H-L-Cross-Adsorbed-Secondary-Antibody-Polyclonal/A-11012)
4. Goat anti-Mouse IgG (H+L) Cross-Adsorbed Secondary Antibody, Alexa Fluor™ 488 (https://www.thermofisher.cn/cn/zh/antibody/product/Goat-anti-Mouse-IgG-H-L-Cross-Adsorbed-Secondary-Antibody-Polyclonal/A-11001)
5. Goat anti-Mouse IgG (H+L) Cross-Adsorbed Secondary Antibody, Alexa Fluor™ 594 (https://www.thermofisher.cn/cn/zh/antibody/product/Goat-anti-Mouse-IgG-H-L-Cross-Adsorbed-Secondary-Antibody-Polyclonal/A-11005)

Eukaryotic cell lines

Policy information about cell lines and Sex and Gender in Research

Cell line source(s): H9C2 cell line was obtained from the American Type Culture Collection (ATCC, CRL-1446).

Authentication: Cell line was only authenticated by the morphology.

Mycoplasma contamination: Cell lines tested negative for mycoplasma.

Commonly misidentified lines (See ICLAC register): None.

Animals and other research organisms

Policy information about studies involving animals; ARRIVE guidelines recommended for reporting animal research, and Sex and Gender in Research

Laboratory animals: C57BL/6C mice (6-8 weeks old, male) were purchased from SLRC Laboratory Animal Co., Ltd. (Shanghai, China). All mice were housed under a specific pathogen-free environment and under standard conditions with 12/12 hours light-dark cycles with free access to water and food. Room temperature was maintained at 20–25°C, and the relative humidity was 40–70%. Newborn Sprague-Dawley rats (1 to 3 days after birth, without discrimination of sex) were purchased from SLRC Laboratory Animal Co., Ltd. (Shanghai, China) for isolating neonatal rat cardiomyocytes.

Wild animals: We didn't use wild animals in our study.

Reporting on sex: To avoid the effect of sex hormones secreted by female mice on the results, only male mice were included in the study. Newborn Sprague-Dawley rats (1 to 3 days after birth) were included without discrimination of sex, during isolation of neonatal rat cardiomyocytes.

Field-collected samples: No field collected samples were used in the study.

Ethics oversight: All animal studies were strictly performed in compliance with the National Institutes of Health (NIH) guidelines and approved by the Animal Care and Use Committee of Shanghai Jiao Tong University.

Note that full information on the approval of the study protocol must also be provided in the manuscript.

Plants

Seed stocks	None.
Novel plant genotypes	None.
Authentication	None.

Flow Cytometry

Plots

Confirm that:

☒ The axis labels state the marker and fluorochrome used (e.g. CD4-FITC).

☒ The axis scales are clearly visible. Include numbers along axes only for bottom left plot of group (a 'group' is an analysis of identical markers).

☒ All plots are contour plots with outliers or pseudocolor plots.

☒ A numerical value for number of cells or percentage (with statistics) is provided.

Methodology

Sample preparation	Cells, underwent OGD/R process, were incubated with the fluorescence probe (DCFH-DA, Beyotime, China) at 37 °C for 20 minutes. After removing the excessive probe, the ROS levels were measured using flow cytometry. For AnnexinV/PI staining, commercial kit (BD Bioscience) was used. Cells with different treatments were digested into single-cell suspension. Staining solution was added into the suspension and incubated for 20 minutes at room temperature in the dark. Samples were assessed on flow cytometer and data was analysed using FlowJov_10.6.1.
Instrument	BD FACSCanto II
Software	FlowJo (version 10.7.1)
Cell population abundance	No cell sorting was performed.
Gating strategy	Cells were circled according to the degree of cell dispersion, and the concentrated clusters were selected for study.

☒ Tick this box to confirm that a figure exemplifying the gating strategy is provided in the Supplementary Information.
