# Supplementary material for: Protective effects of Pt-N-C single-atom nanozymes against myocardial ischemia-reperfusion injury
Source: Nat Commun. 2024 Feb 23;15:1682. doi: 10.1038/s41467-024-45927-3 (PMC10891101; doi:10.1038/s41467-024-45927-3)
Supplement: Supplementary file 3 — Description of Additional Supplementary Files [file 41467_2024_45927_MOESM3_ESM.pdf]

Title: Supplementary Movie 1.
Description: Contractile activity of unstimulated primary NRCMs.

Title: Supplementary Movie 2.
Description: Contractile activity of primary NRCMs incubated with PtsaN-C.

Title: Supplementary Movie 3.
Description: Contractile activity of primary NRCMs after OGD/R injury.

Title: Supplementary Movie 4.
Description: Contractile activity of primary NRCMs treated with PtsaN-C after OGD/R injury.
